# Ribonuclease H2 Subunit A Preserves Genomic Integrity and Promotes Prostate Cancer Progression

**DOI:** 10.1158/2767-9764.CRC-22-0126

**Published:** 2022-08-25

**Authors:** Naoki Kimura, Ken-ichi Takayama, Yuta Yamada, Haruki Kume, Tetsuya Fujimura, Satoshi Inoue

**Affiliations:** 1 Department of Systems Aging Science and Medicine, Tokyo Metropolitan Institute of Gerontology, Tokyo, Japan.; 2 Department of Urology, Graduate School of Medicine, The University of Tokyo, Bunkyo-ku, Tokyo, Japan.; 3 Department of Urology, Jichi Medical University, Tochigi, Japan.; 4 Research Center for Genomic Medicine, Saitama Medical University, Saitama, Japan.

## Abstract

Homeostasis of genomic integrity should be regulated to promote proliferation and inhibit DNA damage–induced cell death in cancer. Ribonuclease H2 (RNase H2) maintains genome stability by controlling DNA:RNA hybrid and R-loop levels. Here, we identified that RNase H2 subunit A (RNASEH2A), a component of RNase H2, is highly expressed in castration-resistant prostate cancer (CRPC) tissues compared with localized prostate cancer. Interestingly, we showed that RNASEH2A suppressed R-loop levels to prevent cell apoptosis induced by DNA damage in prostate cancer cells. Both *in vivo* and *in vitro* studies revealed that RNASEH2A promotes cell growth and migration via the negative regulation of p53 and positive regulation of AR and AR-V7. Mechanistically, epigenetic regulation followed by R-loop accumulation in these promoters was observed for these gene regulations. Importantly, IHC analysis demonstrated that R-loop formation increased in CRPC tissues and correlated with RNASEH2A expression levels. Notably, two small molecules targeting RNase H2 activity were found to suppress CRPC tumor growth with no significant toxic effects. Collectively, we propose that RNASEH2A overexpression is a hallmark of prostate cancer progression by maintaining genomic stability to prevent R-loop–mediated apoptosis induction. Targeting RNase H2 activity could be a potential strategy for treating CRPC tumors.

**Significance:**

RNASEH2A was demonstrated to be highly upregulated in aggressive prostate cancer to degrade R-loop accumulation and preserve genomic stability for tumor growth, suggesting that RNase H2 activity could be a promising therapeutic target.

## Introduction

The maintenance of genomic stability is essential for viable cell activity (1). The formation of DNA:RNA hybrids displaces the other strand in a single-stranded conformation, referred to as an R-loop (2). Active transcription of a locus has been positively associated with DNA:RNA hybrid formation, presumably by the action of transcribed RNA *in cis* or *in trans* and collision with DNA replication (3). The formation of the R-loop has multiple potential consequences for local organization of transcriptional regulatory elements, including double-stranded DNA repair or deleterious effects by increasing genomic instability (3). These structures have also been linked with epigenetic modifications, leading to local effects on nucleic acid conformation changes. DNA:RNA hybrids may be resistant to the activity of DNA methyltransferases (4–6). R-loops have also been reported to stimulate heterochromatin formation by facilitating the compaction of repetitive sequences (5). Furthermore, R-loops interact with various chromatin modulators to regulate the chromatin dynamics (6). Thus, R-loops have multifaceted effects on genome dynamics, depending on their genomic context.

Genome instability is thought to be a hallmark for oncogenesis (1, 7). The formation of double-strand breaks (DSB) activates a cellular response known as the DNA damage response, which recognizes the lesion and coordinates its repair. R-loop accumulation level is controlled by either preventing formation during transcription or degrading already-formed hybrids by RNase H (8, 9). DNA/RNA helicases, such as aquarius (AQR) and senataxin (SETX; ref. 10), and DNA damage components, such as breast cancer 1/2 (BRCA1/2; ref. 11), prevent R-loop–induced DSB formation in human cells. R-loop assembly increases in the absence of factors involved in the maturation or export of mRNAs, including the Transcription-Export (THO/TREX) complex (12). Such assembly is also facilitated by negative supercoiling, which is normally relaxed by topoisomerase (TOP1; ref. 13). The INO80 complex, which contains the INO80 ATPase, prevents replication stress–induced DNA damage and promotes efficient DNA synthesis by counteracting the accumulation of R-loops (8). Thus, these proteins play important roles in the prevention and resolution of R-loop–induced DSBs and viable cancer cell proliferation.

Ribonucleases metabolize RNA, such as breaks in RNA (14). Among the various types of ribonucleases, RNase H hydrolyzes the RNA strand of DNA:RNA hybrids (15). The physiologic function of RNase H is to maintain genome stability by removing primer RNA from Okazaki fragments, R-loop, and ribonucleotide incorporation into DNA (16, 17). Although eukaryote RNase H is classified into type 1 and type 2 (18, 19), RNase H2 exerts major RNase activity and conducts ribonucleotide excision repair in mammals (20). RNase H2 consists of three subunits (A–C) that are necessary and sufficient for RNase H2 activity (20). However, the clinical importance of RNase H2 and its subunits in cancer progression is not fully understood.

Prostate cancer is the most common cancer in men worldwide (21). The mechanisms involved in the development and recurrence of prostate cancer have been investigated. Prostate cancer tumor growth is dependent on the signaling regulated by androgen and its cognate receptor androgen receptor (AR; refs. 22, 23). Although radical prostatectomy or radiation therapy is used to treat localized prostate cancer, androgen deprivation therapy (ADT) is effective for advanced prostate cancer (24). However, most patients with prostate cancer develop castration-resistant prostate cancer (CRPC), a lethal and hormone-refractory phenotype of prostate cancer (22–25). Increased AR signaling at the genomic, epigenetic, and splicing levels has been demonstrated to be essential for the progression to CRPC. Because of the high lethality of patients with CRPC, there is an urgent need to identify gene expression networks and molecular mechanisms responsible for prostate cancer progression.

Previously, we performed RNA-sequence (RNA-seq) analysis to clarify the expression profiles in benign prostate, prostate cancer, and CRPC tissues and identified candidate genes involved in the development of CRPC (23). In the current study, we report that ribonuclease H2 subunit A (RNASEH2A), a conserved catalytic subunit of RNase H2, is one of the genes that are highly upregulated in CRPC tissues compared with benign prostate and prostate cancer tissues. We investigated the clinical implications of RNASEH2A expression in prostate cancer and elucidated the role of RNASEH2A in the progression of CRPC. Notably, we determined the effect of RNase H2 inhibitors (RNase H2 i) on CRPC tumor growth and found that RNase H2 is could have potential efficacy in attenuating prostate cancer progression.

## Material and Methods

### Patient Characteristic and Tissue Preparation

One hundred and six prostatectomy specimens were obtained from radical prostatectomy performed at the University of Tokyo Hospital. Nineteen CRPC specimens were obtained from biopsy, transurethral resection of the prostate (TURP), or radical prostatectomy. Eighteen benign prostate hyperplasia (BPH) cases were obtained from TURP, which was performed at the University of Tokyo (Tokyo, Japan). This study was approved by the institutional ethical committee of the University of Tokyo (#2283) and was conducted in accordance with the Declaration of Helsinki. Written informed consent was obtained from all patients before enrollment.

### Immunostaining and IHC Assessment

IHC analysis for RNASEH2A was performed using the streptavidin-biotin amplification method. Deparaffinized tissue sections were autoclaved (121°C, 10 minutes) in citric acid buffer (2 mmol/L citric acid and 9 mmol/L trisodium citrate dehydrate, pH 6.0) for antigen retrieval. Endogenous peroxidase was inactivated using 0.3% H_2_O_2_. Tissue sections were then incubated in 10% BSA for 30 minutes and then overnight with RNASEH2A antibody (1:50: #16132-1-AP, Proteintech) or S9.6 antibody (1:100, Kerafast). After washing in TBS and 0.1% Tween 20 (TBST), tissue sections were incubated with secondary antibody using CSAⅡ (DAKO). The antigen–antibody complex was visualized with 3,3′-diaminobenzidine tetrachloride (DAB) solution (1 mmol/L DAB, 50 mmol/L Tris-HCl buffer, pH 7.6, and 0.006% H_2_O_2_). At least two independent investigators evaluated the tissue sections by determining the immunoreactivity (IR) score (0, none; 1+, weak; 2+, moderate; 3+, strong signal intensity). RNASEH2A high IR was defined as ≥ 2, and low IR was defined as < 2.

### Cell Culture

We used the prostate cancer cell lines, LNCaP, DU145, PC3, and 22Rv1, as well as the prostate epithelium cell line, RWPE. The cells were purchased from ATCC and grown in RPMI supplemented with 10% FBS, 50 U/mL penicillin, and 50 μg/mL streptomycin. To culture LNCaP cells stably expressing RNASEH2A, 0.5 mg/mL G418 (Nacalai Tesque) was added to the medium. The identity of the cells was confirmed by short tandem repeat analyses in 2019 (BEX Co. Ltd.). We routinely checked for *Mycoplasma* contamination using a PCR-based kit, *Mycoplasma* detection kit (Jena Bioscience). Additional viral infection was checked using PCR method (ICR monitoring center, Kanagawa, Japan). We maintained stocks of low-passage cells and restarted our cell culture with a fresh vial at least once a month. All cell lines were incubated at 37°C in a 5% CO_2_ atmosphere. For androgen treatment, cells were grown in phenol red–free RPMI supplemented with 2.5% charcoal-stripped FBS, 50 U/mL penicillin, and 50 μg/mL streptomycin for 48 hours. We used 10 nmol/L dihydrotestosterone (DHT; Wako) as the androgen stimulation. We purchased docetaxel (DTX) from Sigma, camptothecin (CPT) and etoposide (ET) from Wako. Two RNase H2 is (RNase H2 i #1 and #2) were purchased from Namiki Shoji.

RNase H2 i #1: 2-cyclopentaneamido-4-ethyl-5-methylthiophene-3-carboxa-mide (Supplier: Vitas-M), and RNase H2 i #2: N-[(furan-2-yl)methyl]-2-{8-thia-4,6-diazatricyclo[7.4.0.02,7]trideca-1(9),2(7),3,5-tetraen-3-ylsulfanyl}acetamide (Supplier: OTAVA).

### siRNA

Knockdown of target RNA was performed using Lipofectamine RNAiMAX (Thermo Fisher Scientific). siControl (silencer select siRNA) and sip53 (26), siCENPM, siRNASEH2A, siGINS3, siTRAIP, siSPC24, siKIF23, and siAPEX2 were purchased from Thermo Fisher Scientific. siRNASEH2A (#1 and #2) sequences were designed using siDirect (http://rnai.co.jp/). siRNASEH2As were purchased from RNAi Co., Ltd. The sequences of the siRNAs are as follows. siRNASEH2A # 1: 5′-CAGCAUCCGAGAAUCAGGAGG-3′, 5′-UCCUGAUUCUCGGAUGCUGAG-3′; siRNASEH2A # 2: 5′-GCAGGACUUGGAUACUGAUUA-3′, 5′-AUCAGUAUCCAAGUCCUGCAG-3′.

### Cell Proliferation Assay

Cell proliferation was evaluated by the 3-(4,5-dimethylthiazol-2-yl)-5-(3-carboxymethoxyphenyl)-2-(4-sulfophenyl)2H-tetrazolium, inner salt (MTS) assay using Cell Tier96 (Promega) or counting the number of living cells. For the MTS assay, 3,000 cells were seeded in 96-well plates 24 hours before transfection or DHT/DTX addition. The assays were performed according to the manufacturer's instructions. For cell counting, 5 × 10^4^ cells were seeded in 24-well plates 24 hours before RNase H2 i addition. The cells were stained with 0.5% trypan blue (Nacalai Tesque). Living cells were counted three times.

### Cell Apoptosis Assay

Cells (3 × 10^4^) were seeded on cover glass in 24-well plates 24 hours before transfection, DTX addition, or RNase H2 i addition. After 48 hours of incubation, the DEADEND Fuorometric TUNEL system (Promega) was used to evaluate apoptosis, according to the manufacturer's protocol. 4′,6-diamino-2-phenylindole (Thermo Fisher Scientific) was used to stain the nuclei, and TUNEL-positive cells were observed by confocal laser scanning microscopy (Fluoview FV10I, OLYMPUS). The number of cells was counted in four random fields.

### *In Vivo* Tumor Formation Assay

The basic principles of animal experiments (replacement, reduction, and refinement) were considered in this study. The current study was approved by the institutional ethical committee of the Tokyo Metropolitan Geriatric Hospital and Institute of Gerontology (#0021). LNCaP cells stably expressing RNASEH2A (1.5 × 10^6^) or 22Rv1 (in case of siRNA, cells were used 7.8 × 10^6^; in case of RNase i, 5 × 10^5^ cells) were mixed with Matrigel (BD Biosciences) and injected subcutaneously into BALB/c nude mice. For the siRNA and RNase i experiments, castration was performed after tumor formation. At 24 hours after castration, 5 μg siRNASEH2A or siControl mixed with Lipofectamine RNAimax in OPTI-MEM was injected into each tumor three times per week. RNase i #1 and #2 (5 mg/kg) were injected intraperitoneally twice per week. Tumor volume was calculated using the formula: *V* = 0.5 × *r*1 × *r*2 × *r*3 (*r*1 > *r*2 > *r*3).

### qRT-PCR

Total RNA extracted by ISOGEN (Nippon Gene). First-strand cDNA was generated by Prime Script (Takara). mRNA expressions were measured using Applied Biosystems Step one plus real-time PCR system based on KAPA SYBR Fast kit (Nippon Genetics). mRNA expression levels were calculated by using ΔΔ*C*_t_, adjusted for GAPDH. Primer sequences are listed in Supplementary Table S1.

### DNA:RNA Immunoprecipitation Analysis

DNA:RNA immunoprecipitation (DRIP) was performed as described previously (8, 13). Briefly, DNA was extracted carefully by phenol and/or chloroform extraction, precipitated with ethanol, washed with 70% EtOH, and resuspended in Tris-EDTA (TE) buffer. DNA was then digested with a cocktail of restriction enzymes (HindIII, EcoR1, EcoRV) overnight at 37°C. To obtain samples for RNase H treatment, 10 μg of DNA was treated with 25 U RNase H (Takara) for 5 hours at 37°C. Ten μg of DNA was bound with 10 μL of S9.6 antibody in binding buffer (10 mmol/L NaPO_4_ pH 7, 140 mmol/L NaCl, 0.05% Triton X-100) overnight at 4°C. Protein G-Sepharose beads (GE Healthcare) were added for 2 hours and then washed three times in binding buffer. Elution was performed in elution buffer (50 mmol/L Tris pH 8, 10 mmol/L EDTA, 0.5% SDS, proteinase K) for 1 hour at 55°C. DNA was purified by ethanol precipitation. Furthermore, the fold enrichment relative to input was measured using qPCR with the KAPA SYBR Green PCR master mix (KAPA BIOSYSTEMS) and the ABI StepOne system (Thermo Fisher Scientific). The qPCR primer sequences are listed in Supplementary Table S2.

### DNA:RNA Hybrid Dot Plot Assays

Total nucleic acid was extracted using lysis buffer with SDS/proteinase K, followed by phenol/chloroform extraction and ethanol precipitation. DNA (0.5–1.5 μg) from each sample was spotted on a nylon membrane (Millipore). For RNase H treatment, 1.5 μg of DNA was treated with 10 U RNase H (Takara) at 37°C for 3 hours. Thereafter, the membranes were UV cross-linked, blocked in 5% skim milk/TBST, and incubated overnight at 4°C with mouse S9.6 (1:500). Blots were washed three times with TBST, and secondary antibody (1:10,000 goat anti-mouse horseradish peroxidase) was added for 1 hour at room temperature. The membrane was stained with methylene blue to assess the amount of total DNA.

### Chromatin Immunoprecipitation and Methylated DNA Immunoprecipitation Assay

Chromatin immunoprecipitation (ChIP) and qPCR were performed as described previously (23, 26). In brief, chromatin from cross-linked prostate cancer cells was sonicated, precleared, and incubated with the corresponding antibodies (H3K4me3, K9me2, and K9me3 purchased from Abcam) overnight and precipitated with protein G-sepharose. The DNA–protein antibody complexes were washed with RIPA buffer, Litium buffer, and TE buffer. Cross-linkage of the coprecipitated DNA–protein complexes was reversed, and immunoprecipitated DNA was precipitated with ethanol. Methylated DNA was isolated from 1.0 μg of sonicated DNA using Methylamp Methylated DNA Capture (MeDIP) Kit (Epigentek). In brief, sonicated DNA was added to 5-mC antibody coated well and incubated for 120 minutes at room temperature on an orbital shaker. After releasing with proteinase K for 60 minutes at 65°C, DNA was eluted from the column and adjusted to a final volume of 100 μL with nuclease-free water. The fold enrichment relative to input was measured by performing qPCR using the KAPA SYBR Green PCR master mix and the ABI StepOne system (Thermo Fisher Scientific). Primer sequences are listed in Supplementary Table S2.

### Statistical Analysis

We used Excel (Microsoft) and JMP Pro version 15.0 (Analytics) for statistical analysis. The log-rank test was used to assess survival. Univariate and multiple Cox proportional models were used to evaluate independent predictors of PSA-free survival in patients with prostate cancer. Pearson *χ*^2^ test was performed to analyze the association between RNASEH2A IR and clinicopathologic parameters. Two-sided Student *t* test or two-way analysis of variance was performed for functional analysis. Statistical significance was defined as *P* values less than 0.05.

### Data Availability

RNA-seq data have been deposited in the Japanese Genotype-phenotype Archive (JGA) under accession code JGAS00000000198. Publicly available microarray data was obtained using Oncomine (https://www.oncomine.org). Other relevant data in this study are available from the corresponding author upon reasonable request.

## Results

### Identification of *RNASEH2A* as a Frequently Upregulated Gene in CRPC Tissues

**FIGURE 1:**
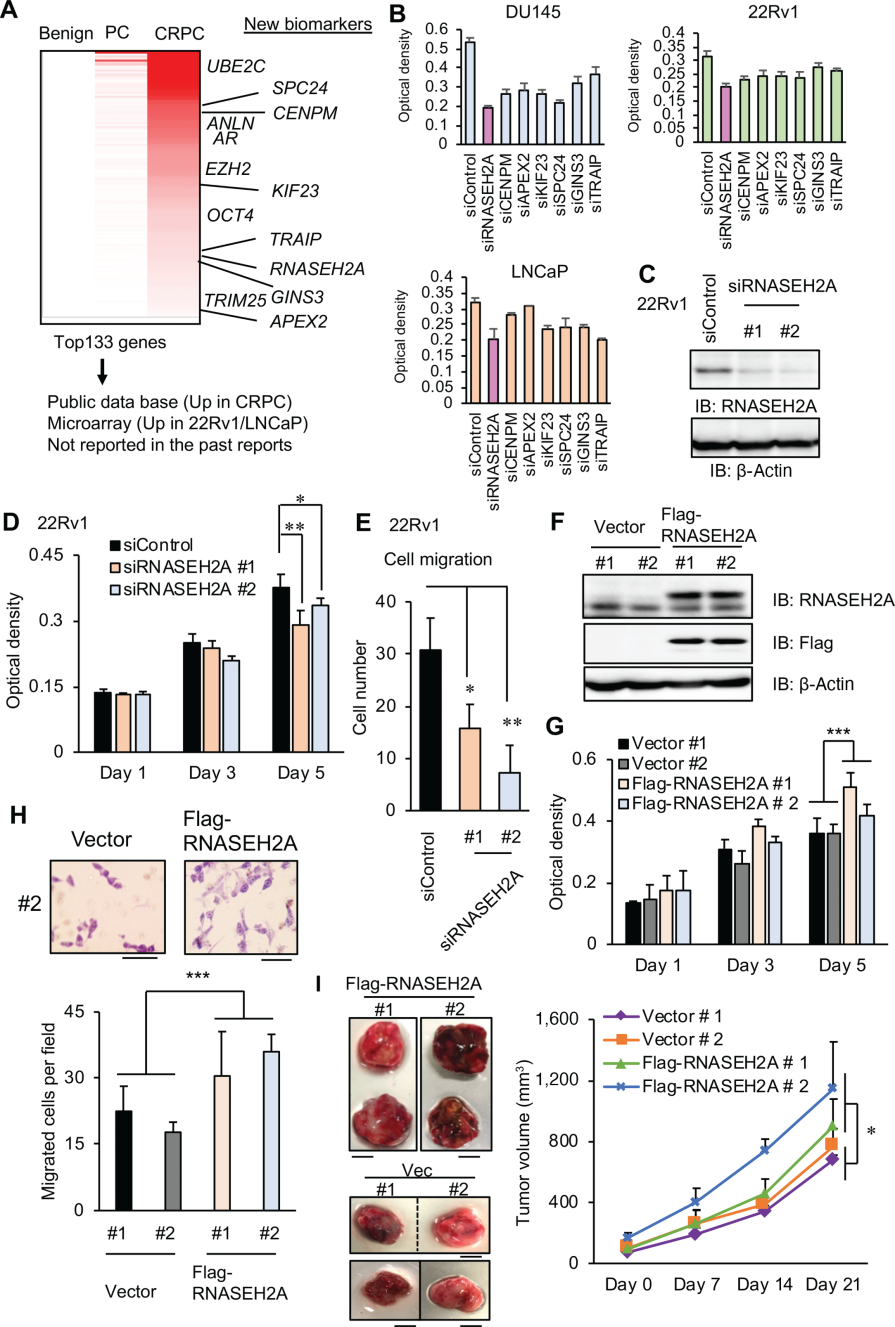
Identification of RNASEH2A that contributes to the aggressiveness of prostate cancer **A,** Heatmap of gene expression in benign prostate, primary prostate cancer, and CRPC tissues. Average RNA-seq results (RPKM) are shown. We selected candidate genes among significantly upregulated genes in CRPC which met the presented conditions shown in the panel. **B,** MTS assay of prostate cancer cells transfected with siControl or siRNA targeting each candidate gene (*N* = 4). **C,** Knockdown of RNASEH2A expression by two siRNAs. Western blot analysis was performed in 22Rv1 cells. **D,** MTS assay was performed in 22Rv1 treated with siRNASEH2A #1, #2 or siControl (*N* = 6). **E,** Migrated cell number was counted in siRNASEH2A #1, #2 treated 22RV1. Statistical analysis was performed by using the two-sided Student *t* test (*, *P* < 0.05; **, *P* < 0.01). Data are presented as the mean ± SD. **F,** Stable overexpression of RNASEH2A expression in LNCaP cells. Western blot analysis was performed to evaluate the expression level. IB: immunoblot. **G,** MTS assay was performed in RNASEH2A stably expressing LNCaP and vector control (*N* = 6). **H,** Top, Representative migrated cell picture of vector and RNASEH2A stably expressing cells. Bar = 10 μm. Bottom, Migrated cell number of RNASEH2A or vector stably expressing cells. Statistical analysis was performed by using two-way ANOVA (*, *P* < 0.05). Data are presented as the mean ± SD. **I,** RNASEH2A or vector stably expressing LNCaP was injected subcutaneously into nude mice. Left, Pictures of representative two tumors injected with RNASEH2A or vector stably expressing LNCaP. Bar = 5 mm. Right, Tumor growth in nude mice (*N* = 4). Statistical analysis was performed by using two-way ANOVA (*, *P* < 0.05). Data are presented as the mean ± SE.

To identify the genes responsible for prostate cancer progression, we analyzed the gene expression profiles in our RNA-seq dataset containing benign prostate, primary prostate cancer, and CRPC samples (23). A subset of genes was found to be upregulated in CRPC tissues compared with prostate cancer tissues (Fig. 1A). Among them, we selected genes that were confirmed to be upregulated in CRPC tissues in other cohorts or CRPC model cells using public microarray or RNA-seq database. After excluding previously reported genes, we identified seven candidates responsible for CRPC development (*RNASEH2A, CENPM, APEX2, KIF23, SPC24, GINS3*, and *TRAIP*). To evaluate the functional role of these candidates, we performed a loss-of-function assay with several prostate cancer cells, including CRPC model cells such as 22Rv1 and DU145 cells (Fig. 1B). Among them, we observed the most marked repression of cell growth when the expression of RNASEH2A was suppressed. Thus, we focused on the role of RNASEH2A in advanced prostate cancer.

To further determine the effect of RNASEH2A on the aggressiveness of prostate cancer cells, we performed loss-of-function assays using two independent siRNAs in prostate cancer cells (Fig. 1C; Supplementary Fig. S1A). Accordingly, knockdown of RNASEH2A was shown to decrease cell growth and migrating cell number compared with siControl (Fig. 1D and E; Supplementary Fig. S1B and S1C). Consistently, repression of the cell-cycle markers, cyclin D1 and cyclin E, was observed following RNASEH2A knockdown (Supplementary Fig. S1D). In contrast, the overexpression of RNASEH2A in LNCaP cells promoted cell proliferation and migration compared with the control (Fig. 1F–H). Moreover, RNASEH2A overexpression accelerated tumor growth *in vivo* compared with the control (Fig. 1I; Supplementary Fig. S1E). Taken together, these results indicate the important role of RNASEH2A in the aggressiveness of prostate cancer tumors.

### Loss of RNASEH2A Leads to DNA Damage and Apoptosis

**FIGURE 2:**
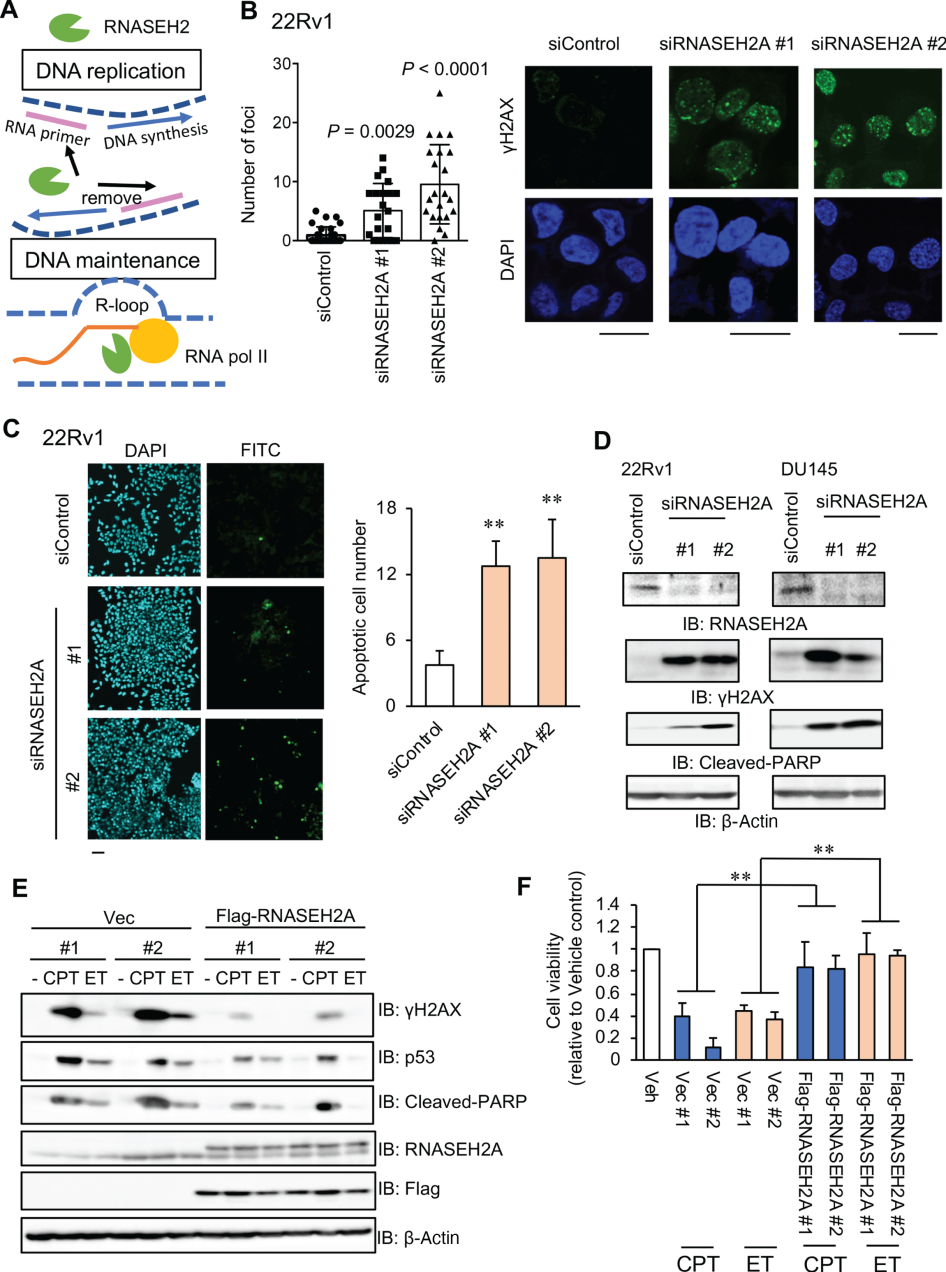
RNASEH2A inhibits DNA damage response and apoptosis in prostate cancer cells. **A,** Schematic summary of RNase H2 in human cells. **B,** Immunofluorescence images of DNA damage marker p-γH2AX. 22Rv1 was treated with siRNASEH2A #1, #2, or siControl. Bar = 10 μmol/L. Statistical analysis was performed using the two-sided Student *t* test. **C,** Left, Induction of apoptosis by RNASEH2A knockdown. 22Rv1 were treated with siRNASEH2A #1, #2 or, siControl. Apoptotic cells were detected by TUNEL assay. Representative images of FITC positive cells treated with siRNASEH2A #1, #2 or siControl for 48 hours. Bar = 50 μm (Right) Quantification of TUNEL positive cell (*N* = 4). Statistical analysis was performed by using the two-sided Student *t* test (**, *P* < 0.01). Data are presented as the mean ± SD. **D,** Immunoblots of p-γH2AX, RNASEH2A, and apoptosis marker cleaved-PARP in DU145 and 22Rv1 cells. **E,** LNCaP cells overexpressing Flag-RNASEH2A or vector control were treated with 0.5 μmol/L CPT or 10 μmol/L ET for 24 hours. Western blot analysis was performed to evaluate the expression level of indicated proteins. IB: immunoblot. **F,** MTS assay was performed in LNCaP cells overexpressing Flag-RNASEH2A or vector control were treated with 0.03 μmol/L CPT or 3 μmol/L ET for 72 hours. Cell viability relative to vehicle-treated control for each cell group is shown. Statistical analysis was performed by two-way ANOVA (**, *P* < 0.01). Data are presented as the mean ± SD.

RNASEH2A is a component of RNase H2 and is responsible for enzymatic activity (15–17). RNase H2 functions to eliminate R-loop accumulation which leads to DNA damage and promotes DNA replication by degrading Okazaki fragments. Thus, RNaseH2 has a key role in the stabilization of genomic status for cancer cell growth (Fig. 2A). First, we explored the role of RNASEH2A in maintaining genomic integrity in prostate cancer cells by affecting RNase H2 activity (17). Briefly, cells were treated with siRNASEH2A and DNA damage responses were assessed using immunofluorescence and Western blotting for γH2A, a marker of DNA damage signaling. Knockdown of RNASEH2A in prostate cancer cells led to increased nuclear γH2A immunostaining (Fig. 2B; Supplementary Fig. S2A and S2B). This enhanced DNA damage corresponded to increased apoptosis in prostate cancer cells treated with siRNASEH2A (Fig. 2C; Supplementary Fig. S2C and S2D). Conversely, repressed apoptosis was found when it was induced by the anticancer reagent, DTX, in RNASEH2A-overexpressing LNCaP cells (Supplementary Fig. S2E and S2F). RNASEH2A silencing was also shown to increase the cleavage of PARP, an indicator of apoptosis, and γH2A expression by Western blot analysis (Fig. 2D).

We further examined whether RNASEH2A overexpression confers resistance to DSB-induced cell apoptosis using chemotherapy drugs which cause DNA DSBs [camptothecin (CPT) and etoposide (ET), topo isomerase inhibitors]. We observed that inhibition of cell viability, induction of apoptosis and DNA breaks were alleviated by overexpressing RNASEH2A (Fig. 2E and F). Taken together, these data suggest the preventive role of RNASEH2A in DNA damage signaling and apoptosis in CRPC cells.

### RNASEH2A Regulates the Expression Levels of p53 and AR to Promote Tumor Growth

**FIGURE 3:**
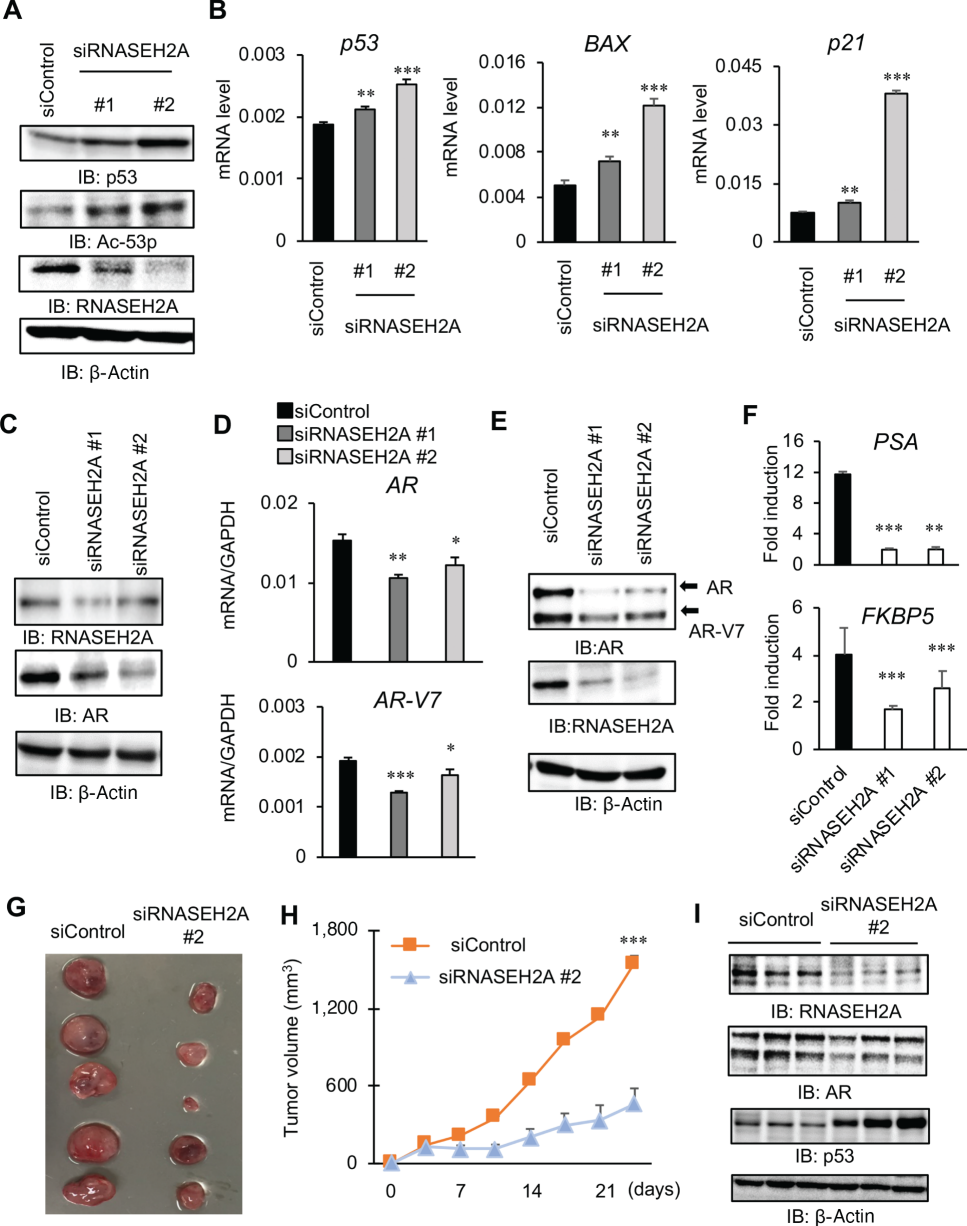
RNASEH2A regulates p53 and AR signaling to promote CRPC tumor growth. **A,** 22Rv1 was treated with siRNASEH2A #1, #2, or siControl. RNASEH2A, p53, and acetylated-p53 (Ac-p53) protein levels were evaluated by Western blot analysis. **B,** Measurement of *p53* and p53 downstream (*BAX* and *p21*) mRNA expression. Statistical analysis was performed by using the two-sided Student *t* test (**, *P* < 0.01; ***, *P* < 0.001). Data are presented as the mean ± SD. **C,** AR protein expression in LNCaP cells treated with siRNASEH2A. Protein expression was evaluated by Western blot analysis. **D** and **E,** mRNA and protein expressions of AR and AR-V7 in siRNASEH2A-treated 22Rv1. mRNA expression was measured by qRT-PCR, and protein expression was evaluated by Western blot analysis. **F,** Androgen-induced transcription was alleviated by RNASEH2A knockdown. LNCaP cells were treated with vehicle or 10 nmol/L DHT for 24 hours. Cells were treated with siRNASEH2A #1, #2 or siControl 24 hours before the hormone treatment. qRT-PCR analysis was performed to determine mRNA expression level (*N* = 3). Fold induction by DHT treatment was calculated. Statistical analysis was performed by using of two-sided Student *t* test (*, *P* < 0.05; **, *P* < 0.01). Data are presented as the mean ± SD. **G**–**I,** CRPC tumor growth was attenuated by RNASEH2A knockdown. Nude mice were injected subcutaneously with 22Rv1 cells. Castration was performed in all mice after tumor formation. Then, siRNASEH2A or siControl was injected into the tumor. **G,** Representative picture of siControl or siRNASEH2A treatment mice. Bar = 10 mm. **H,** Tumor volume shift in nude mice was illustrated (*N* = 10). Statistical analysis was performed by using the two-sided Student *t* test (***, *P* < 0.001). Data are presented as the mean ± SE. **I,** RNASEH2A, AR, and p53 expressions in resected tumor from mice were evaluated by using Western blot analysis. IB: immunoblot.

Dysregulation of p53 expression and activity is frequently observed in CRPC tissues (27). An increase in p53 expression levels in response to DNA damage leads to the activation of many genes that trigger apoptosis. Therefore, we focused on the correlation between the expression of RNASEH2A and p53. First, knockdown of RNASEH2A was found to increase the expression of p53 (Fig. 3A; Supplementary Fig. S3A). Furthermore, knockdown of RNASEH2A increased the mRNA expression of *p53*, *BAX*, and *p21* compared with siControl (Fig. 3B; Supplementary Fig. S3B). We previously reported that p53 expression was induced by DTX treatment (27). On the basis of the MTS assay, LNCaP cells stably expressing RNASEH2A maintained the advantage of cell growth compared with the control vector in the presence of DTX (Supplementary Fig. S3C). In addition, Western blot analysis showed that DTX induction of p53 was attenuated in LNCaP cells overexpressing RNASEH2A compared with control cells (Supplementary Fig. S3D and S3E), suggesting a negative role of RNASEH2A in p53 expression.

AR is the central signal for prostate cancer progression, and CRPC development is caused by enhanced AR-mediated epigenetic control or gene induction (22–25). Therefore, we assessed whether RNASEH2A affects the expression or downstream signals of AR. Knockdown of RNASEH2A decreased the expression of AR and its variant, AR-V7 (Fig. 3C–E; Supplementary Fig. S4A). Furthermore, RNASEH2A knockdown decreased the mRNA levels of AR downstream genes (*FKBP5*, *ACSL3*, *TRIM36*, and *PSA*; Fig. 3F; Supplementary Fig. S4B). We also observed these gene regulations by RNASEH2 in prostate cancer cells in androgen-depleted medium (Supplementary Fig. S4C). Consistently, DHT-dependent upregulation of prostate cancer cell growth was significantly abrogated by RNASH2A knockdown (Supplementary Fig. S5A). In contrast, the overexpression of RNASEH2A resulted in increased expression of AR and its target, *PSA* (Supplementary Fig. S5B–S5D), as well as enhancement of cell growth depending on DHT compared with the control (Supplementary Fig. S5E). To further validate the role of RNASEH2A knockdown *in vivo*, nude mice were injected subcutaneously with 22Rv1 cells. After tumor formation, all mice were castrated, and the siControl or siRNASEH2A was injected into the tumor. siRNAEH2A treatment significantly inhibited tumor growth compared with siControl (Fig. 3G and H). As expected, the expression of AR and AR-V7 was alleviated, and p53 expression was enhanced in RNASEH2A reduced tumor (Fig. 3I). Consistent with a previous study where p53 was found to repress AR expression in LNCaP cells (28), we observed that p53 knockdown induced AR expression (Supplementary Fig. S6). Collectively, these results suggest that RNASEH2A regulates p53 and AR signaling to promote CRPC tumor growth.

### Epigenetic Regulations by RNASEH2A Loss Involves R-Loop Accumulation at the Promoters

**FIGURE 4:**
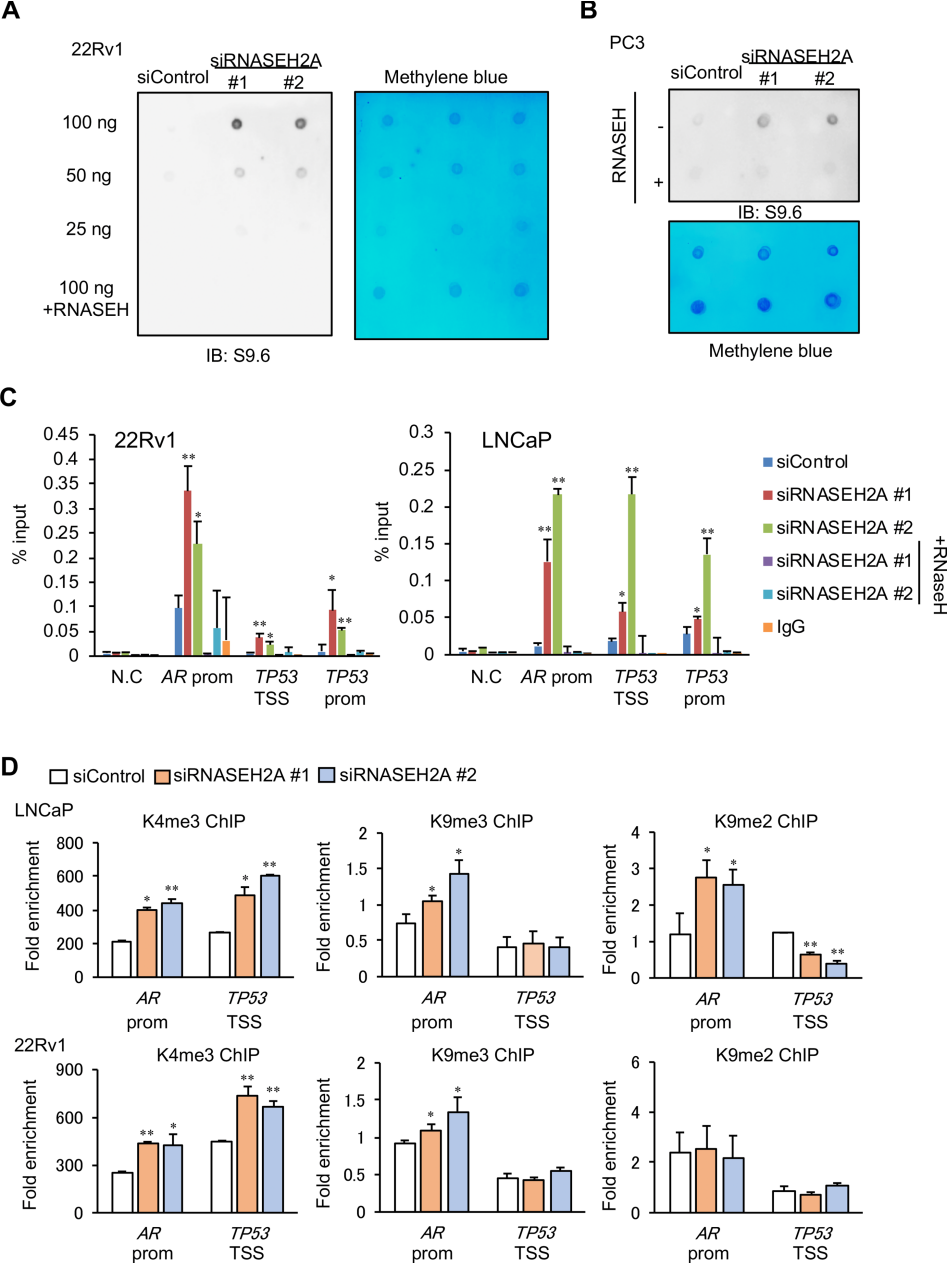
RNASEH2A repressed R-loop accumulation around *TP53* and *AR* promoter regions. **A,** Dot plot analysis to evaluate R-loop accumulation. DNA obtained from 22Rv1 cells treated with siRNASEH2A #1, #2, or siControl for 48 hours was used. Immunoblot with S9.6 antibody was performed. Methylene blue staining is shown to show the total DNA amount. Recombinant RNASEH was used to analyze the specificity to DNA:RNA hybrids. **B,** Dot plot analysis to evaluate R-loop accumulation. DNA obtained from PC3 cells treated with siRNASEH2A #1, #2, or siControl for 48 hours was used. **C,** DRIP assay around *TP53* and *AR* promoter regions. 22Rv1 and LNCaP cells were treated with siRNASEH2A #1, #2, or siControl for 48 hours. DRIP by S9.6 was performed. Recombinant RNASEH was used to analyze the specificity to DNA:RNA hybrids. Fold enrichment relative to input sample was measured by qPCR. N.C: negative control, prom: promoter, TSS: transcriptional start site. **D,** ChIP assay of H3K4me3, H3K9me2, and H3K9me3 was performed (*N* = 3). 22Rv1 and LNCaP cells were treated with siControl, siRNASEH2A #1, or #2 for 48 hours. Statistical analysis was performed by using of two-sided Student *t* test (*, *P* < 0.05; **, *P* < 0.01). Data are presented as the mean ± SD.

We determined whether R-loops affect gene regulation and DNA-damage response caused by RNASEH2A knockdown. To evaluate whether RNASEH2A regulates the levels of R-loop accumulation, dot plot analysis was performed using S9.6, which specifically detects DNA:RNA hybrids as shown in previous reports (6–9). We observed that increased S9.6 signals were observed following RNASEH2A knockdown (Fig. 4A and B). These S9.6 signals were eliminated by exogenous RNase H addition *in vitro*, suggesting the specificity of this antibody. To determine whether RNASEH2A prevents the accumulation of R-loops formed at specific genomic loci, a DRIP assay (8, 13) was conducted using prostate cancer cells treated with siControl or siRNASEH2A (Fig. 4C). Significant enrichment of DNA/RNA hybrids was found at the promoter regions of *p53* and *AR* in RNASEH2A-depleted prostate cancer cells compared with the control. Importantly, the elevated levels of DNA/RNA hybrids by RNASEH2A inhibition were reduced by treatment with recombinant RNase H prior to DRIP. Thus, these results suggest that RNASEH2A suppresses the accumulation of R-loops formed at these gene promoters.

As R-loops are enriched at CpG islands, several past studies reported recognition and interaction with specific epigenetic modifiers (4–6). We proceeded to determine whether RNASEH2A affects the epigenetic conditions of these promoter regions. Briefly, the ChIP assay was performed to assess the epigenetic modification by trimethylation of lysine 4 of histone H3 (H3K4me3), an active histone marker, and dimethylation or trimethylation of lysine 9 of histone H3 (H3K9me2 and H3K9me3), which are repressive histone markers at these genomic loci (Fig. 4D). qPCR revealed increased H3K4me3 at both the *TP53* and *AR* promoters by knockdown of RNASEH2A. However, specifically upregulated H3k9me2 and H3K9me3 levels were observed only in the *AR* promoter region. In addition, we analyzed DNA methylation (5-mC: 5-methyalated cytosine) levels around these CpG islands. However, significant induction of DNA methylation was not observed by suppressing RNASEH2A (Supplementary Fig. S7). These results indicate that histone modification changes on the promoter regions would be associated with gene regulation by RNASEH2A.

### Clinical Significance of RNASEH2A and R-Loop Accumulation in Lethal CRPC Tissues

**FIGURE 5:**
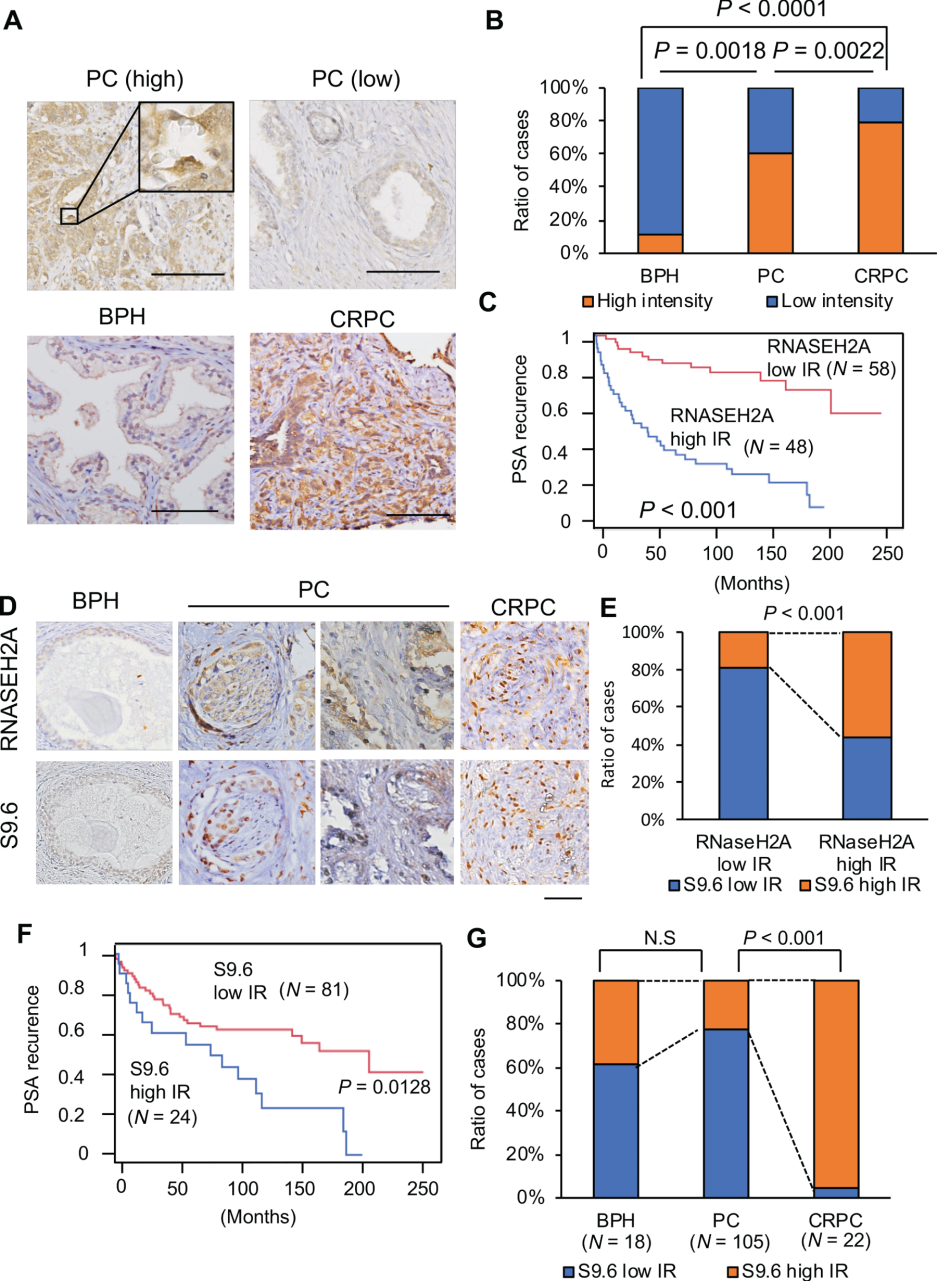
High expression of RNASEH2A is observed in CRPC tissues at protein level and correlated with R-loop accumulation. **A–C,** IHC RNASEH2A expression in prostate cancer specimens. **A,** Representative images of RNASEH2A in specimens of benign prostate, prostate cancer and CRPC tissues. Bar = 100 μmol/L. **B,** The ratio of RNASEH2A high IR in BPH, prostate cancer, and CRPC tissues is shown. Statistical analysis was performed by *χ*^2^ test. **C,** Kaplan–Meier curve of progression-free survival in prostate cancer (*N* = 106). **D**–**G,** IHC analysis of R-loop accumulation using S9.6 antibody in prostate cancer specimens. **D,** Representative images of R-loop in specimens of BPH, prostate cancer, and CRPC tissues. Bar = 100 μm. **E,** The expression level of RNASEH2 protein detected by IHC correlated with S9.6 IR in prostate cancer tissues. **F**, Kaplan–Meier curve of progression-free survival in prostate cancer (*N* = 106). **G,** The ratio of S9.6 high IR in BPH, prostate cancer, and CRPC tissues is shown. Statistical analysis was performed by *χ*^2^ test.

Counteracting R-loops promotes cancer proliferation and repressive effects on DNA damage–induced apoptosis in cancer cell lines (8, 13). How R-loops are clinically involved in the aggressiveness of prostate cancer remains unclear. To clarify this point, sections of benign, prostate cancer, and lethal CRPC tissues were used in an IHC analysis of RNASEH2A and R-loops. RNASEH2A immunostaining was detected in both the nuclei and cytoplasm of the prostate cancer tissues (Fig. 5A). Consistent with the results of RNA-seq analysis, the proportion of high IR of RNASEH2A was significantly enriched in aggressive patients with prostate cancer (high Gleason score, pT stage, and pN stage) (Supplementary Table S3). The IR of RNASEH2A was higher in CRPC tissues than localized prostate cancer tissues (Fig. 5B). Kaplan–Meier curves showed that high RNASEH2A IR was significantly associated with poor survival compared with low RNASEH2A IR (Fig. 5C, *P* < 0.001; Supplementary Table S4). Importantly, only RNASEH2A among all subunits of RNase H2 (H2A, H2B, and H2C) was upregulated in CRPC tissues in other cohorts at the mRNA level (Supplementary Fig. S8), suggesting the importance of RNASEH2A in RNAseH2 activity in CRPC. We analyzed the R-loop accumulation in prostate cancer samples by assessing the S9.6 signals. Notably, a significant increase in R-loop signals was found in CRPC tissues compared with benign and localized prostate cancer tissues (Fig. 5D). Interestingly, R-loop accumulation was significantly associated with the expression level of RNASEH2A (Fig. 5E) in prostate cancer tissues and poor outcome of patients with prostate cancer and prostate cancer progression (Fig. 5F and G), suggesting a dysregulated R-loop accumulation during prostate cancer progression and subsequent increase in RNASEH2A. Taken together, these clinical findings are consistent with our model in which stress accumulation during cancer progression was assumed to drive R-loop accumulation, which is further attenuated by RNASEH2A induction to prevent R-loop–mediated DNA damage and apoptosis.

### Inhibition of RNase H2 Activity is Effective for Suppressing CRPC Tumor Growth

**FIGURE 6:**
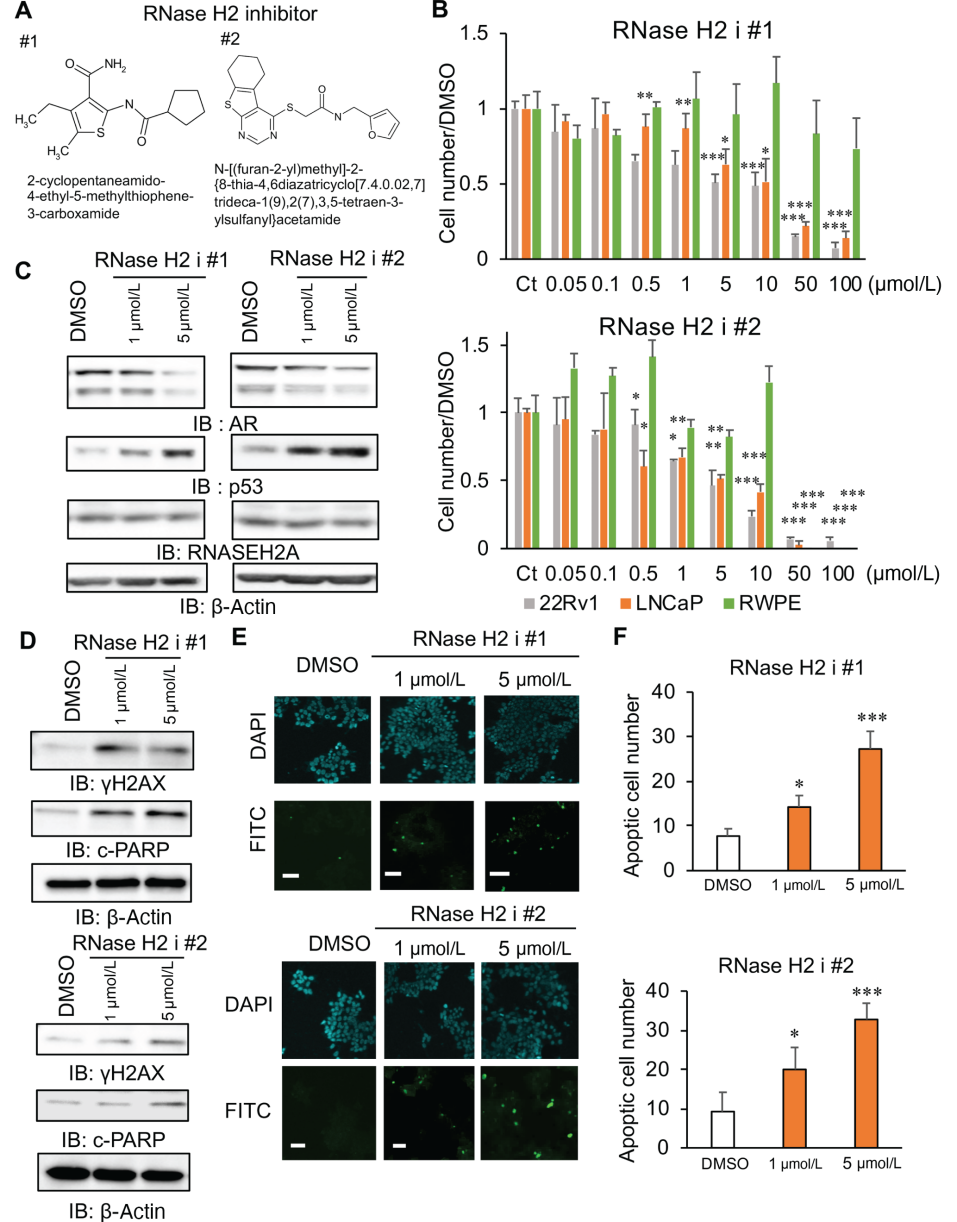
Treatment with RNase H2 i suppressed prostate cancer cell growth via downregulation of AR and upregulation of p53. **A,** Chemical structures of RNase H2 i #1 and #2. **B,** The effect of RNase H2 i treatment on prostate cancer cell growth. Prostate cancer (LNCaP and 22Rv1) and benign prostate (RWPE) cells were treated with RNase H2 i #1, #2 or vehicle for 72 hours. Viable cell numbers were counted by staining with trypan blue. Ct: vehicle control. Statistical analysis was performed by using the two-sided Student *t* test (*, *P* < 0.05; **, *P* < 0.01; ***, *P* < 0.001). Data are presented as the mean ± SD. **C,** Western blot analysis of the expression of AR, p53, and RNASEH2A. 22Rv1 cells were treated with RNase H2 i #1, #2 or vehicle for 48 hours. **D,** Immunoblots of p-γH2AX and cleaved-PARP in 22Rv1 cells treated with RNase H2 i #1, #2 or vehicle for 48 hours. IB: immunoblot. **E,** Representative images of FITC positive cells treated with RNase H2 i #1, #2 or vehicle for 48 hours. **F,** Quantification of TUNEL positive cell (*N* = 4). Statistical analysis was performed by using of two-sided Student *t* test (*, *P* < 0.05; ***, *P* < 0.001). Data are presented as the mean ± SD.

**FIGURE 7:**
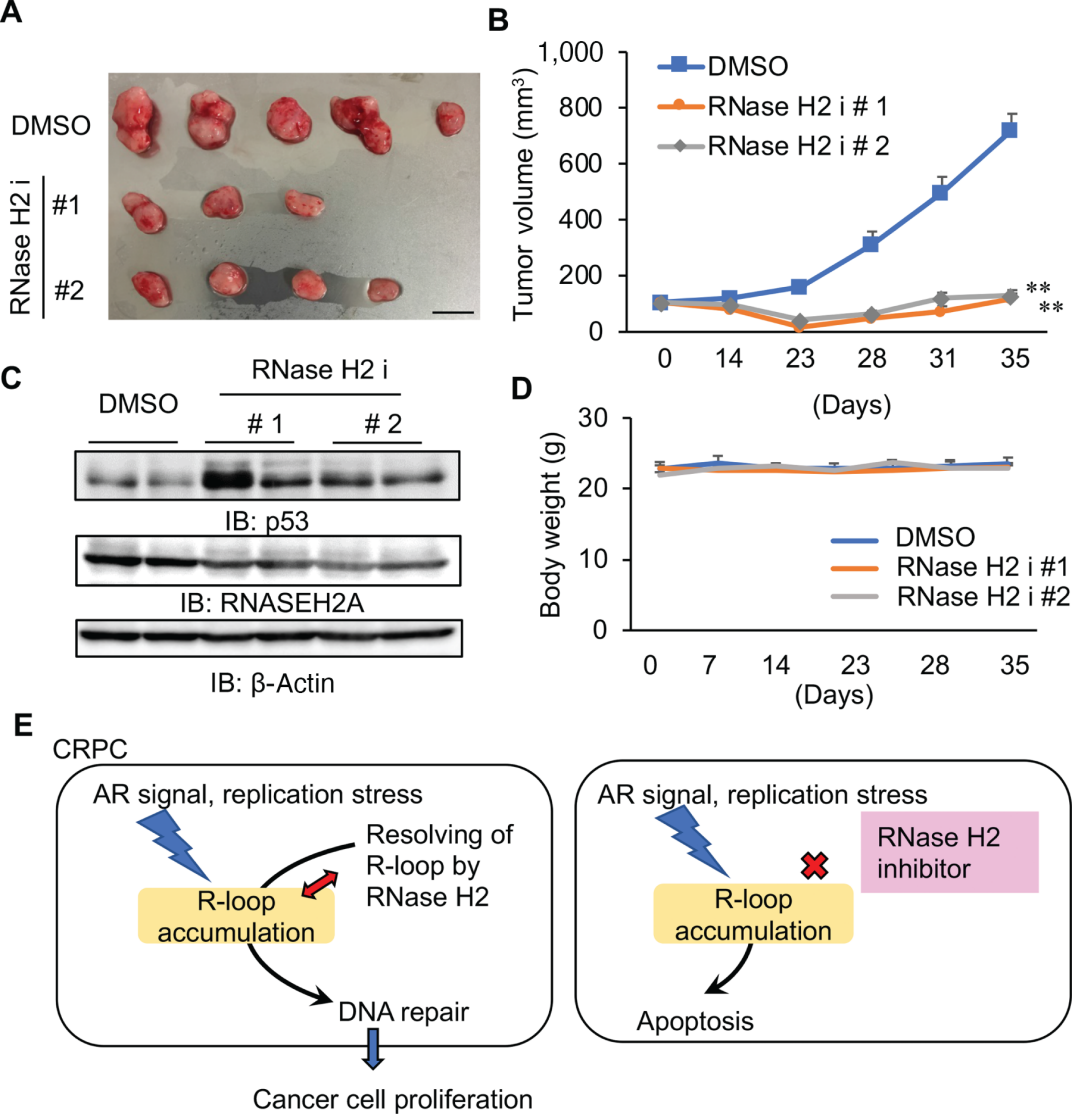
Pharmacologic inhibition of RNase H2 activity impairs CRPC tumor growth. **A–D,** Administration of RNase H2 i have an inhibitory effect in CRPC tumor growth. Nude mice were injected subcutaneously with 22Rv1 cells. Mice were castrated after tumor development. Then, RNase H2 i # 1, # 2 or vehicle (DMSO) was injected intraperitoneally. **A,** Representative images of vehicle, RNase H2 i # 1, or # 2 treated tumors. Bar = 10 mm. Two tumors of RNase H2 i #1 and one tumor of RNase H2 i #2 disappeared with the treatment. **B,** Growth of tumors in nude mice are shown (*N* = 5). Statistical analysis was performed by using the two-sided Student *t* test (**, *P* < 0.01). Data are presented as the mean ± SE. **C,** RNASEH2A and p53 expression in tumors were evaluated by Western blot analysis. DMSO: dimethyl sulfoxide, IB: immunoblot. **D,** Body weight of mice is not significantly affected by administration of RNase H2 i (#1 and #2) compared with vehicle control. **E,** Schematic diagram of RNASEH2A in prostate cancer progression. Blockade of RNase H2 activity could be a promising therapeutic strategy for suppressing CRPC growth.

Recently, RNase H inhibitors have been developed to prevent the propagation of human immunodeficiency virus by removing primer RNA using RNase H activity of the host cells during viral replication (29, 30). As RNASEH2A is essential for RNase H2 activity, we used two small molecules that specifically inhibit RNase H2 activity (RNase H2 i # 1, # 2; Fig. 6A) and explored the possibility that RNase H2 is an effective agent for treating CRPC. First, we observed that treatment with RNase H2 i # 1 and # 2 inhibited the growth of 22Rv1 and LNCaP cells. Meanwhile, a high concentration of RNase H2 was required to suppress the growth of the benign prostate cells, RWPE cells (Fig. 6B). Thereafter, we determined the effects of RNase H2 on the expression of AR, p53, γH2AX, and c-PARP in prostate cancer cells (Fig 6C and D; Supplementary Fig. S9A and S9B). RNase H2 i treatment was demonstrated to downregulate AR and AR-V7 expression (Fig. 6C) and upregulate γH2AX expression in prostate cancer cells (Fig. 6D; Supplementary Fig. S9B). Consequently, p53 expression was upregulated by RNase H2 i (Fig. 6C; Supplementary Fig. S9A), which led to increased apoptosis of prostate cancer cells (Fig. 6E and F; Supplementary Fig. S9C and S9D). Notably, we demonstrated the anticancer effect of RNaseH2 i *in vivo*. In xenograft models of AR-positive 22Rv1, we performed castration to inhibit androgen action, thereby mimicking hormone therapy. The marked inhibition of castration-resistant tumor growth was observed in RNase H2 i-treated mice compared with the vehicle control (Fig. 7A and B). The protein expression of p53 in tumors was increased in mice injected with RNase H2 (Fig. 7C). Furthermore, no apparent tissue failure or body weight loss was observed (Fig. 7D; Supplementary Fig. S10). Taken together, our observations revealed the growth inhibitory effects for alleviating the aggressiveness of CRPC upon inhibition of RNase H2 activity (Fig. 7E).

## Discussion

In this study, we demonstrated that the overexpression of RNASEH2A is one of the mechanisms that accelerate castration resistance in prostate cancer. RNase H2, the major isoform of RNase H enzyme that degrades DNA/RNA hybrids, is a heterotrimeric complex composed of a conserved catalytic subunit, RNASEH2A, and auxiliary subunits RNASEH2B and RNASEH2C (31). The physiologic role of human RNase H2 subunits has been elucidated by the observation that mutations in any of its subunits were found to cause Aicardi-Goutieres syndrome, a neuroinflammatory disease associated with the chronic activation of the immune system to an excessive accumulation of aberrant forms of nucleic acids (32, 33). Importantly, our clinicopathologic analysis showed that RNASEH2A is highly expressed in CRPC tissues at the protein level compared with localized prostate cancer. Notably, high expression of RNASEH2A in prostate cancer tissues predicts poor prognosis in patients with prostate cancer. Both *in vitro* and *in vivo* studies revealed that RNASEH2A promotes CRPC tumor growth and prevents DNA damage–mediated apoptosis. Taken together, these observations indicate that high expression of RNASEH2A is an exacerbation factor for prostate cancer. Consistent with our findings, high expression of RNASEH2A may be involved in various types of cancers (34–36). Interestingly, the expression of RNASEH2A was found to be upregulated in CRPC, while that of RNASEH2B was downregulated in prostate cancer according to the publicly available database (Supplementary Fig. S6), as observed in a previous study (37). Deletion of RNASEH2B decreased p53 expression in the development of intestinal tumors in embryonic mice (38). In contrast, the overexpression of RNASEH2A repressed p53 expression. Therefore, we expect that the specific regulation of individual RNase H2 subunit expression may be important for prostate cancer progression.

Importantly, our study revealed that R-loops were robustly enriched around the *TP53* and *AR* promoter regions by inhibiting RNASEH2A expression. Furthermore, dot plot analysis showed that R-loop accumulation in prostate cancer cells was promoted by RNASEH2A silencing, suggesting the effect of RNASEH2A expression on RNase H2 activation. Consistent with our results, knockdown of RNASEH2A was reported to decrease the activity of RNase H2 in breast cancer (32). The R-loop is known to function as a transcriptional activator or repressor at specific loci by various mechanisms, including epigenetic regulation (3, 39, 40). RNASEH2A negatively regulates the expression of p53 in prostate cancer. Meanwhile, knockout of RNASEH2A activates p53 downstream signals in mice (38, 19). These results indicate that the increased p53 expression by knockdown of RNASEH2A in prostate cancer is caused by the inactivation of RNase H2, followed by the induction of genome instability. Moreover, RNASEH2A was found to positively regulate AR and AR-V7 expression. The expression of AR-V7 in CRPC correlates with resistance to chemotherapy, such as DTX and cabazitaxel (41, 42). Therefore, we can speculate that the repression of AR signals by suppressing RNASEH2A expression may improve the efficiency of ADT and chemotherapy. Our experimental results by p53 knockdown suggest that the repressive effect of RNASEH2A on p53 expression could enhance the expression level of AR, as expected from a previous study (28). Furthermore, as another mechanism, our analysis demonstrated that histone H3K4me3, an active epigenetic marker, around these promoter regions was induced by RNASEH2A knockdown. These results could reflect those of past studies showing the overlap between R-loop accumulation and activated histone marks (6, 39, 40). However, in *AR* promoter regions, enrichment of the repressive histone marks, H3K9me2 and H3K9me3, increased via RNASEH2A depletion, which might be involved in the repression of *AR* transcription. In other reports, the persistence of a subset of R-loops can impair the expression of specific genes (5, 6). Although it should be further investigated whether histone modifications are regulated by R-loop accumulation, we speculate that DNA hybridization with transcribed RNA in active transcriptional regions may induce pausing of transcription, thereby repressing the transcription process or negatively affecting epigenetic condition. Interestingly, bidirectional promoters are used for the transcription of *TP53* and its antisense RNA (43). There might be additional mechanisms that the antisense RNA transcripts would be involved in R-loop formation to regulate transcription and epigenetic conditions.

IHC analysis revealed that enhanced accumulation of R-loops was markedly observed in CRPC specimens compared with localized prostate cancer and benign prostate tissue. To our knowledge, this is the first study to reveal the clinicopathologic significance of R-loops in clinical cancer samples. R-loops could occur due to various stresses or stimulations in cancer cells (44, 45). Multiple replication origins are reported to encounter chromatin regions occupied by transcription machinery in proliferating cancer cells, causing transcription replication conflicts and collision events (5, 13, 44). Such collision events can induce the formation of R-loops. Thus, R-loop formation is highly abundant due to replication stress, leading to DNA damage and genome instability. In addition, DNA/RNA hybrids accumulate in the proximity of DSBs in transcriptionally active genomic regions (11, 45). R-loops also serve as a key structure within transcriptionally active regions to facilitate the accurate repair of DSBs (10, 46). Sex steroid hormones are reported to be involved in the formation of R-loops in cancer (47, 48). Estrogen promotes breast cancer–associated translocations by stimulating R-loop formation at target genes during the S-phase in breast cancer cells (47). Androgen regulates *EWSR1* breakpoint formation by inducing R-loop accumulation for DNA damage in prostate cancer (48). These transcription by-products are a major threat to genome integrity as they are prone to DNA breakage. Therefore, we speculate that these cellular stresses would contribute to the enrichment of R-loops in CRPC tumors.

Once formed, DNA/RNA hybrids can be degraded by RNase H (9) or resolved by specific helicases (10–12). In addition, inhibition of topoisomerase enhanced R-loop formation and DSBs in cancer cells (13). We showed that topoisomerase inhibitors (CPT or ET) treatment-mediated inhibition of cell viability, induction of apoptosis and DNA breaks were alleviated by overexpression of RNASEH2A. Herein, we present the first evidence that the high expression of RNASEH2A promotes CRPC tumor growth. We propose that a mechanism to prevent R-loop–mediated genome instability is necessary for cancer progression. Past evidence also suggests that failure to resolve R-loops by inhibiting some of these factors and subsequent induction of DNA breaks have detrimental effects on cancer cell proliferation and homeostasis (8, 13). Thus, enrichment of R-loops in chromatin induces genomic instability and cell apoptosis. Another study reported that DNA repair genes regulated by androgen are highly expressed in CRPC tissues (49), suggesting the importance of preserving genomic integrity in the aggressiveness of prostate cancer. Collectively, we present a promising model in which excessive genomic instability due to R-loop accumulation could be suppressed by inducing RNASEH2A expression in CRPC to promote tumor growth.

Notably, we demonstrated that the inactivation of RNase H2 activity could be a potential strategy for treating CRPC. We used two small molecules that function as RNase H2 is. These inhibitors specifically suppress RNase H2 activity without affecting RNase H1 activity (29, 30). We observed that p53 was upregulated while AR and AR-V7 was downregulated in prostate cancer cells treated with these two inhibitors, indicating that the repressive effect of RNase H2 activity is responsible for gene regulation. These findings are in line with the notion that the effect of RNASEH2A knockdown on prostate cancer cells occurs via the inhibition of RNase H2 activity. Moreover, RNase H2 is were found to induce the expression of γH2AX, a DNA damage marker, as well as apoptosis. Therefore, the repression of RNase H2 activity could be a promising strategy for advanced prostate cancer therapy by increasing sensitivity to chemotherapy and DNA damage–induced apoptosis.

## Supplementary Material

Supplementary Methods, Tables S1-S4 Supplementary Table S1. PCR primer sequences for RT-PCR. Supplementary Table S2. Primer sequences for ChIP/DRIP assay. Supplementary Table S3. Relationships of RNASEH2A immunoreactivity (IR) score with clinicopathological findings in prostate cancer (PC) patients. Supplementary Table S4. Univariate and multivariate analysis for recurrence free survival in PC patients.

Supplementary Figures S1-S10 Supplementary Figure S1. Loss of RNASEH2A expression repressed CRPC cell growth and migration. Supplementary Figure S2. RNASEH2A inhibits DNA damage response and apoptosis in PC cells.
Supplementary Figure S3. siRNASEH2A promotes p53 expression in LNCaP. Supplementary Figure S4. RNASEH2A positively regulates AR expression and downstream signaling. Supplementary Figure S5. RNASEH2A positively regulates AR-mediated cell growth. Supplementary Figure S6. Knockdown of p53 induced AR expression and cell growth. Supplementary Figure S7. DNA methylation is not significantly affected by RNASEH2A knockdown.
Supplementary Figure S8. Each RNASEH2 subunit gene expression in PC by using public database. Supplementary Figure S9. The effect of RNase H2 i treatment on LNCaP cells. Supplementary Figure S10. No apparent toxic effect was observed in tissues of mice treated with RNase H2 i.
