# Supplementary material for: Ribonuclease H2 Subunit A Preserves Genomic Integrity and Promotes Prostate Cancer Progression
Source: Cancer Res Commun. 2022 Aug 25;2(8):870–83. doi: 10.1158/2767-9764.CRC-22-0126 (PMC10010380; doi:10.1158/2767-9764.CRC-22-0126)
Supplement: Supplementary Methods, Tables S1-S4 — Supplementary Table S1. PCR primer sequences for RT-PCR. Supplementary Table S2. Primer sequences for ChIP/DRIP assay. Supplementary Table S3. Relationships of RNASEH2A immunoreactivity (IR) score with clinicopathological findings in prostate cancer (PC) patients. Supplementary Table S4. Univariate and multivariate analysis for recurrence free survival in PC patients. [file crc-22-0126-s01.docx]

**Supplementary methods**

**Cell migration assay**

Cell migration assay was performed by transwell migration assay. Cell culture inserts with 8.0 µm pore size PET filter (Corning, N.Y, USA) were incubated in phosphate buffer saline (PBS) containing 1% fibronectin (Sigma-Aldrich、St.Louis、USA) for 30 minutes. 5×10^4^ cells were seeded in the insert and incubated at 37℃ 5%CO_2_ for 24h. The cells on the upper surface were wiped with a cotton swab. Then the filters were soaked in methanol for 30 minutes, washed with PBS, and stained with Giemsa. After washing with PBS, the filters were mounted on slide glasses. We observed migrated cells on the lower surface by optical microscope and counted cell numbers in four random fields. Data were evaluated by calculating mean value ± S.D.

**Plasmid construction and stably expressing cell establishment**

Human RNASEH2A was amplified by polymerase chain reaction (PCR), and inserted into N-terminal Flag-tagged pcDNA3 (Thermofisher). An expression vector containing Flag-tagged human RNASEH2A cDNA and expression vector alone were transfected into LNCaP cells according to the manufacture’s protocol. Transfected cells were cultured in RPMI medium containing 0.5 mg/mL G418, and it was employed as stably transfected LNCaP cells.

**Western blotting analysis**

Protein extracts prepared by NP40 buffer (50mM Tris, pH 8.0, 150 mM NaCl, 1% NP-40) containing protease inhibitor (Nacalai tesque, Kyoto, Japan). Protein concentrations were measured by the BCA protein assay kit (Thermo Fisher). The same amounts of proteins were resolved by 10% polyacrylamide gel electrophoresis and electroblotted onto Immobilon-P membrane (Merck Millipore, Darmstadt, Germany). Membranes were incubated with primary antibody of anti-RNASEH2A (Cat#ab55990, Abcam, Cambridge, UK), anti-AR (H-280, Santa Cruz Biotechnology, California, USA), anti-p53 (Cat#sc-126, Santa Cruz Biotechnology), anti-acetylated-p53 (Cat#2525, Cell signaling), anti-Cyclin D1(Cat#sc-246, Santa Cruz Biotechnology), anti-Cyclin E (Cat#sc-247, Santa Cruz Biotechnology), anti-γH2AX (Cat # ab11174, Abcam)、anti-cleaved-PARP (Cat # ab32064, Abcam) and anti-β-actin (Wako, Osaka, Japan) antibody diluted in 1:2000, 1:2000, 1:200, 1:1000, 1:500, 1:200, 1:2000, 1:5000, 1:2000, respectively. Following secondary antibody incubation, antibody-antigen complexes were detected by Pierce ECL Plus (ThermoFisher).

**Supplementary Table S1. PCR primer sequences for RT-PCR**

*FKBP5* Fw: 5’-CTGCAGAGATGTGGCATTCACT-3’

Rv: 5’-TCCAGAGCTTTGTCAATTCCAA-3’

*AR* Fw: 5’-TGTGGAAGCTGCAAGGTCTTC-3’

Rv: 5’-TCTGCTGGCGCACAGGTA-3’

*GAPDH* Fw: 5’-GGTGGTCTCCTCTGACTTCAACA-3’

Rv: 5’-GTGGTCGTTGAGGGCAATG-3’

*AR-V7* Fw: 5’-CAGGGATGACTCTGGGAGAA-3’

Rv: 5’-GCCCTCTAGAGCCCTCATTT-3’

*PSA* Fw: 5’-CAGGAACAAAAGCGTGATCTTG-3’

Rv: 5’-GCTGTGCTGACCTGAAATACC-3’

*ACSL3* Fw: 5’-TGCTTTCCGAAGCTGCTATT-3’

Rv: 5’-AAGGCATCTGTCACCAGACC-3’

*TRIM36* Fw: 5’-AGTTCTGGAAGAGAGGAAATC-3’

Rv: 5’-TCCATTTGAGTCTGAAATTG-3’

*p53* Fw: 5’-CCCCTCGAGTCAGGAAACA-3’

Rv: 5’-TCATCTGGACCTGGGTCTTC-3’

*p21* Fw: 5’-GGAAGACCATGTGGACCTGT-3’

Rv: 5’-GGATTAGGGCTTCCTCTTGG-3’

*BAX* Fw: 5’-GTCGCCCTTTTCTACTTTGC-3’

Rv: 5’-CTCAGCCCATCTTCTTCCAG-3’

**Supplementary Table S2. Primer sequences for ChIP/DRIP assay**

N.C Fw: CCTGGAGGGCTTGGAGATG

Rv: GATCCTACGGCTGGCTGTGA

*AR* promoter Fw: GGAGGCGACAGAGGGAAAAA

Rv: ACCAGGCACTTTCCTTGCTT

*TP53* TSS Fw: AGCGATTTTCCCGAGCTGAA

Rv: TGCTCAAGACTGGCGCTAAA

*TP53* promoter Fw: GGCAAAAAGAAACCGAAAT

Rv: GCTGTCAGTCGTGGAAGTGA

*TP53* upstream Fw: CTAGGGCTTGATGGGAACGG

Rv: GGATCCGACGCAGAGCTAAA

**Supplementary Table S3. Relationships of RNASEH2A immunoreactivity (IR) score with clinicopathological findings in prostate cancer (PC) patients**

RNASEH2A IR score (N = 106)

Intensity < 2 (N = 63) Intensity ≥ 2 (N = 43) *P*-value

Age (±SD) 　　　　 　　67.4±6.0　　　 　　65.9±5.8 0.20

Serum PSA

PSA < 10 ng/mL 35 20 0.434

PSA ≥ 10 ng mL 28 22

Gleason score (GS)

GS < 8 45 19 **0.0082**

GS ≥ 8 18 24

Pathological T stage

pT < 3b 46 25 0.141

pT ≥ 3b 17 18

Pathological N stage

pN = 0 58 35 0.133

pN ≥ 1 5 8

IR score was evaluated by using the intensity (0, none; 1, weak; 2, moderate; and 3, strong). Note that serum PSA was not measured in one patient. Two-sided Student’s t-test and Fisher’s exact test was used for statistical analysis. GS; Gleason score, PSA: prostate specific antigen, SD: standard deviation

**Supplementary Table S4. Univariate and multivariate analysis for recurrence free survival in PC patients**

Univariate　 Multivariate

Parameters HR (95％CI) *P-*value　 HR (95%CI) *P-*value

Serum PSA 0.83 (0.47 - 1.4) 0.52

(PSA < 10 ng/mL vs ≥ 10 ng/mL)

Gleason score (GS) 0.26 (0.15 - 0.47) **< 0.001** 0.45 (0.22 - 0.93) 0.093

(GS < 8 vs ≥ 8)

Pathological T stage 0.29 (0.17 - 0.51) **< 0.001** 0.41 (0.19 - 0.89) **0.0035**

(pT < 3b vs ≥ 3b)

Pathological N stage 0.38 (0.19 - 0.75) 　**0.0061** 1.77 (0.78 - 4.0) 0.268

(N0 vs ≥ N1)

RNASEH2A IR 0.19 (0.10 - 0.36） **< 0.001** 0.19 (0.099 - 0.38) **< 0.001**

(IR < 2 vs IR ≥ 2)

Cox proportional hazard model were used for univariate and multivariate analysis. *P*-value of < 0.05 was considered to be statistically significant. CI; confidence interval, HR; hazard ratio. PSA: prostate specific antigen,
