# Supplementary material for: Ribonuclease H2 Subunit A Preserves Genomic Integrity and Promotes Prostate Cancer Progression
Source: Cancer Res Commun. 2022 Aug 25;2(8):870–83. doi: 10.1158/2767-9764.CRC-22-0126 (PMC10010380; doi:10.1158/2767-9764.CRC-22-0126)
Supplement: Supplementary Figures S1-S10 — Supplementary Figure S1. Loss of RNASEH2A expression repressed CRPC cell growth and migration. Supplementary Figure S2. RNASEH2A inhibits DNA damage response and apoptosis in PC cells. Supplementary Figure S3. siRNASEH2A promotes p53 expression in LNCaP. Supplementary Figure S4. RNASEH2A positively regulates AR expression and downstream signaling. Supplementary Figure S5. RNASEH2A positively regulates AR-mediated cell growth. Supplementary Figure S6. Knockdown of p53 induced AR expression and cell growth. Supplementary Figure S7. DNA methylation is not significantly affected by RNASEH2A knockdown. Supplementary Figure S8. Each RNASEH2 subunit gene expression in PC by using public database. Supplementary Figure S9. The effect of RNase H2 i treatment on LNCaP cells. Supplementary Figure S10. No apparent toxic effect was observed in tissues of mice treated with RNase H2 i. [file crc-22-0126-s02.pdf]

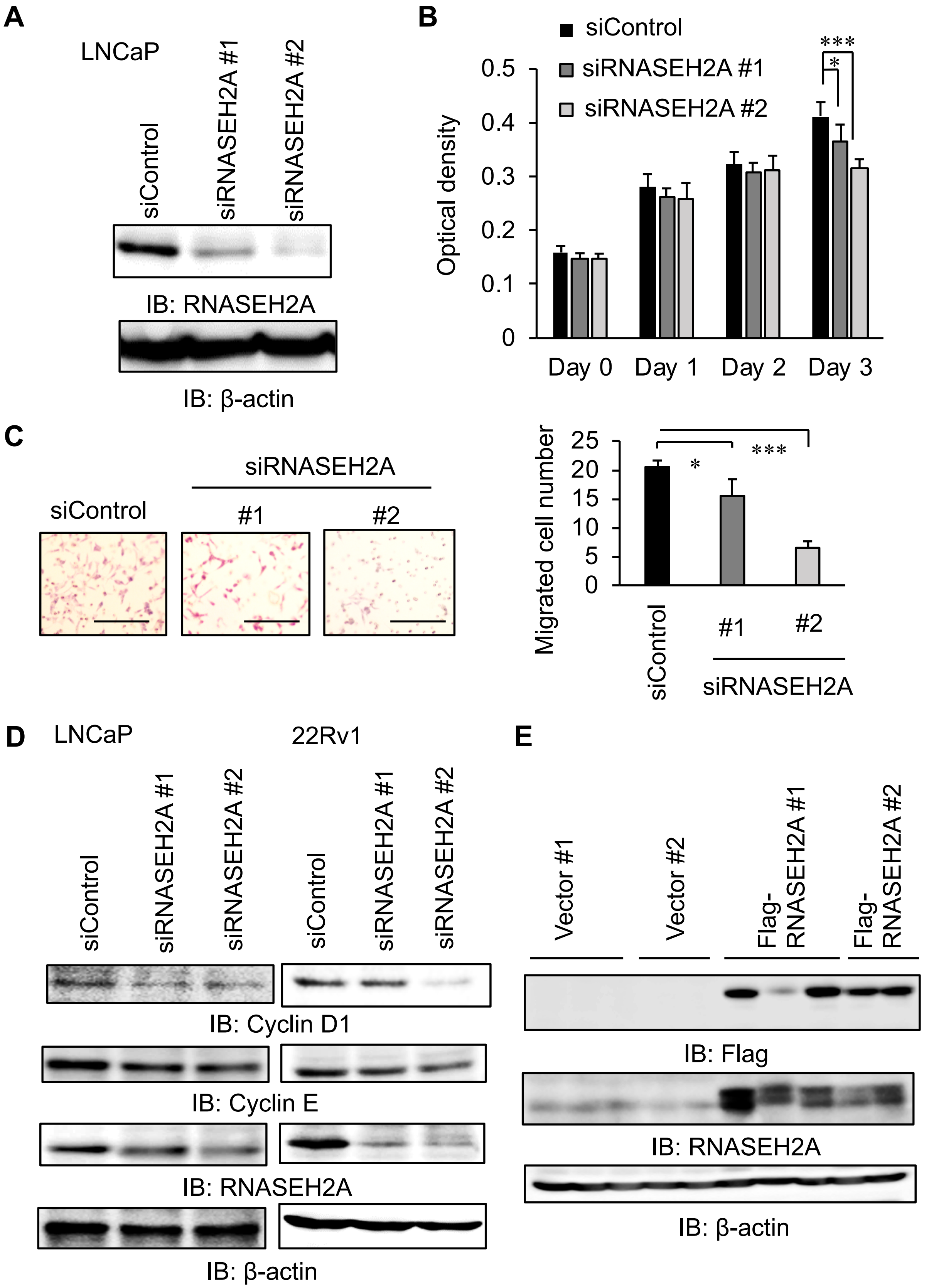

Supplementary Figure S1. Loss of RNASEH2A expression repressed CRPC cell growth and migration. (A) The protein level of RNASEH2A was evaluated in LNCaP treated with siRNASEH2A #1, #2 or siControl by western blot analysis. (B) MTS assay was performed in LNCaP treated with siRNASEH2A #1, #2 or siControl. Statistical analysis was performed by using the two-sided Student's t-test (*: $P < 0.05$, **: $P < 0.01$). Data represent mean + SD (N = 6). (C) (Left) Representative migrated cell picture of LNCaP treated with siControl or siRNASEH2A #1, # 2. Bar = 10 µm. (Right) Migrated cell number was counted in siRNASEH2A #1, #2 treated LNCaP. Statistical analysis was performed by using the two-sided Student's t-test (*: $P < 0.05$, **: $P < 0.01$). Data represent mean + SD (N = 4). (D) Evaluation of the effect of RNASEH2A knockdown on the protein level of Cyclin D1 and Cyclin E by western blot analysis. (E) The protein level of RNASEH2A was evaluated in resected tumors from nude mice by western blot analysis.

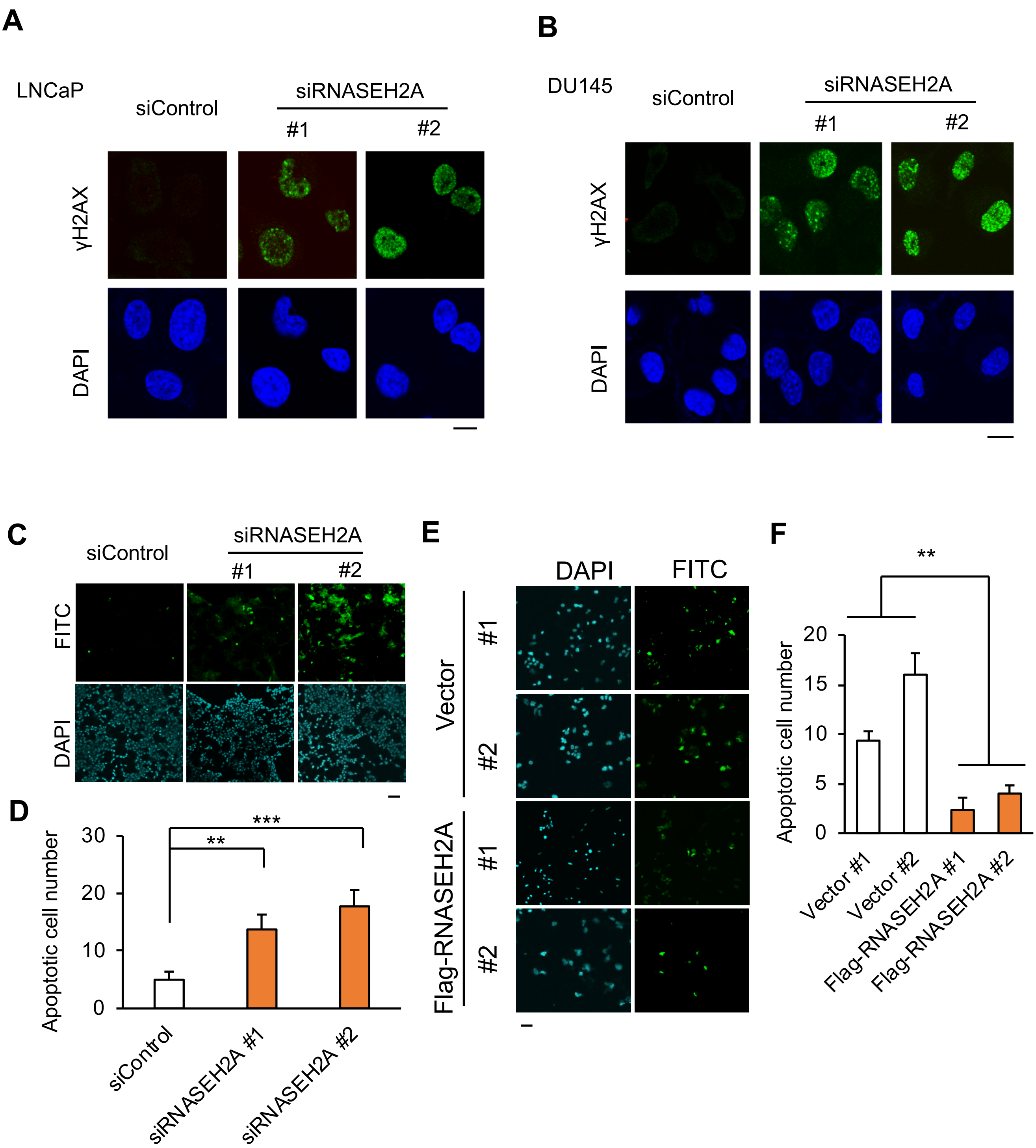

Supplementary Figure S2. RNASEH2A inhibits DNA damage response and apoptosis in PC cells. (A, B) Immunofluorescence (IF) images of DNA damage marker γH2AX. LNCaP (A) and DU145 (B) cells were treated with siRNASEH2A #1, #2, or siControl. Bar = 10μm. (C) Induction of apoptosis by RNASEH2A knockdown. LNCaP cells were treated with siRNASEH2A #1, #2 or, siControl. Representative images of FITC positive cells treated with siRNASEH2A #1, #2 or siControl for 48 h. Bar = 10μm. (D) Quantification of TUNEL positive cell. Statistical analysis was performed by using the two-sided Student's t-test (**: $P < 0.01$, ***: $P < 0.001$). Data represent mean + SD (N=4). (E) RNASEH2A stably expressing LNCaP or vector control treated with docetaxel (DTX) 0.2 nM. Representative images of FITC positive cells were shown. Bar = 10μm. (F) Quantification of TUNEL positive cell. Statistical analysis was performed by using Two-way ANOVA and two-sided Student's t-test (**: $P < 0.01$). Data represent mean + SD (N=4).

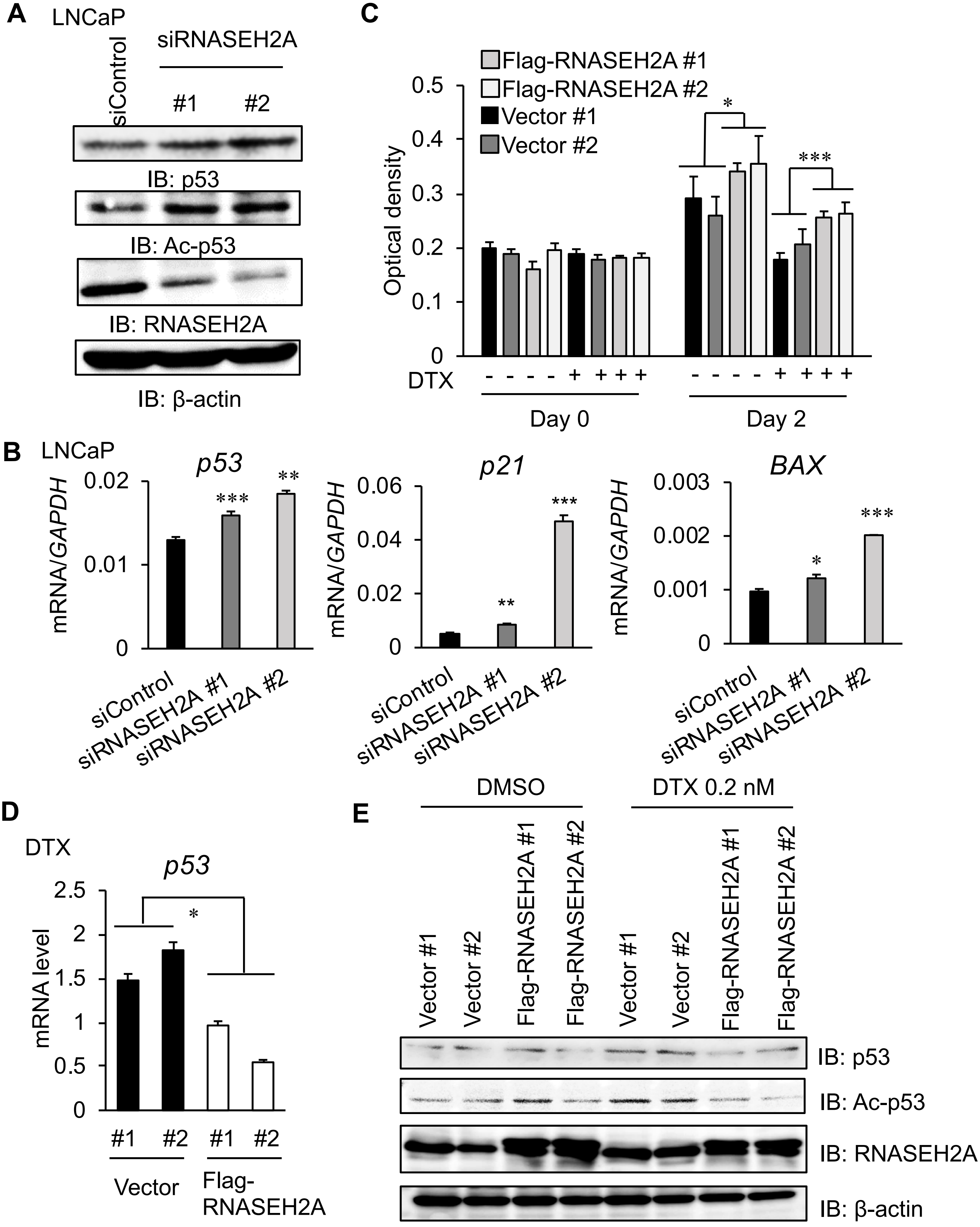

Supplementary Figure S3. siRNASEH2A promotes p53 expression in LNCaP. (A. B) LNCaP was treated with siRNASEH2A #1, #2, or siControl. (A) RNASEH2A, p53, and acetylated-p53 (Ac-p53) protein levels were evaluated by western blot analysis. (B) Measurement of *p53* and p53 downstream (*BAX* and *p21*) mRNA expression. Statistical analysis was performed by using the two-sided Student's t-test (**: $P < 0.01$, ***: $P < 0.001$). Data represent mean + SD (N = 3). (C) Cell proliferation assay of LNCaP cells stably expressing RNASEH2A or vector control with or without the treatment of DTX 0.2 nM. Statistical analysis was performed by using of two-way ANOVA (*: $P < 0.05$, ***: $P < 0.001$). Data represent mean + SD (N = 6). (D, E) p53 was induced by treatment of DTX 0.2 nM in LNCaP cells stably expressing RNASEH2A and vector control. (D) Evaluation of DTX induced mRNA level of *p53*. Statistical analysis was performed by using two-way ANOVA (*: $P < 0.05$). Data represent mean + SD (N = 3). (E) DTX induced p53 and acetylated-p53 (Ac-p53) protein level were evaluated by western blot analysis.

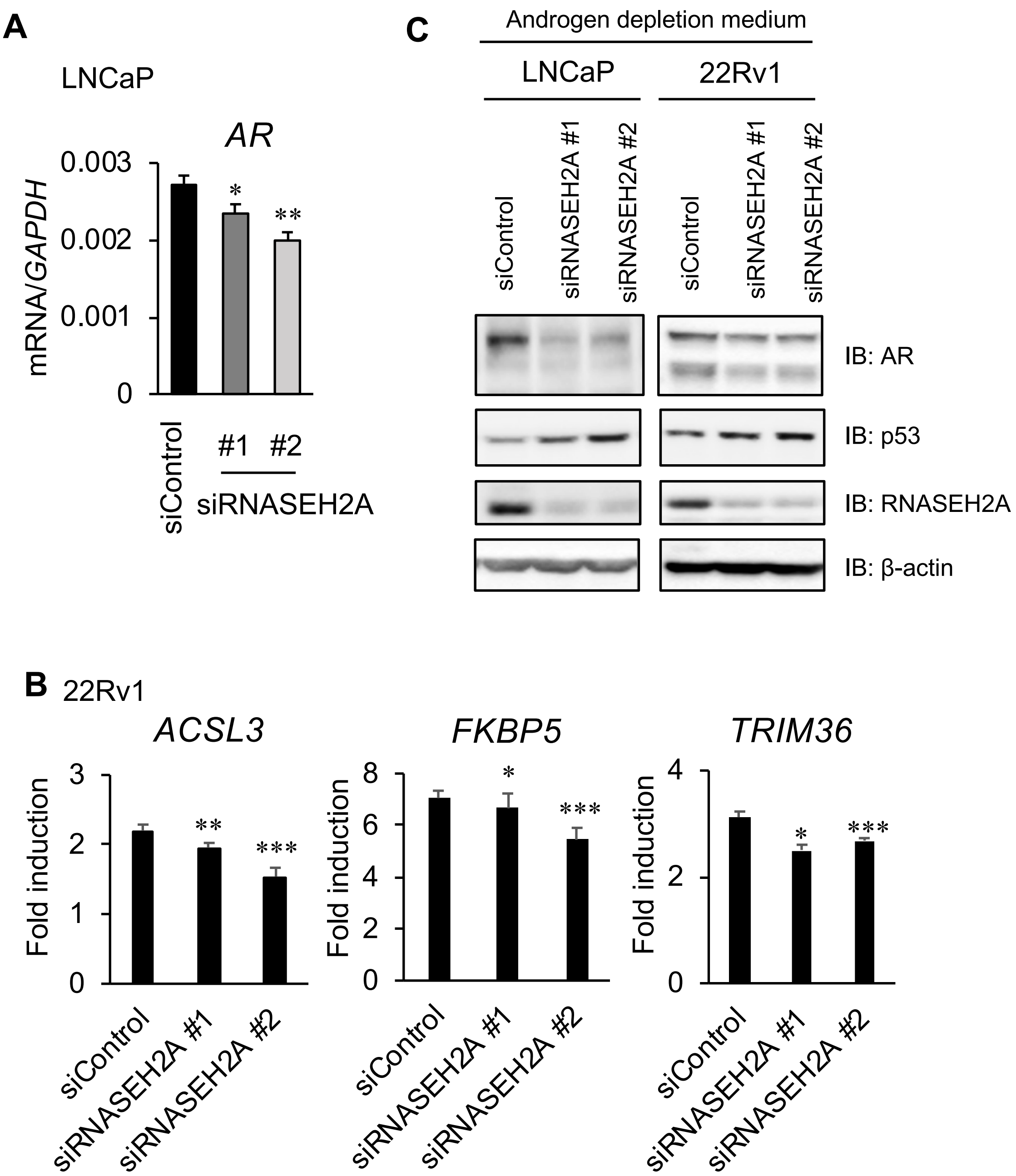

Supplementary Figure S4. RNASEH2A positively regulates AR expression and downstream signaling. (A) mRNA expression of AR in siRNASEH2A treated LNCaP. mRNA expression was measured by qRT-PCR Statistical analysis was performed by using of two-sided Student's t-test (*: $P < 0.05$, **: $P < 0.01$). Data represent mean + SD (N = 3). (B) Androgen-induced transcription was alleviated by RNASEH2A knockdown. 22Rv1 cells were treated with vehicle or 10 nM DHT for 48 h. Cells were treated with siRNASEH2A #1, #2 or siControl 24 h before the hormone treatment. qRT-PCR analysis was performed to determine mRNA expression level. Fold induction by DHT treatment was calculated. Statistical analysis was performed by using of two-sided Student's t-test (*: $P < 0.05$, **: $P < 0.01$). Data represent mean + SD (N = 3). (C) Protein expressions of AR and p53 in siRNASEH2A treated LNCaP and 22Rv1 cells in the absence of androgen. Cells were incubated in androgen-depleted medium (Phenol-red free RPMI medium including 2.5 % charcoal stripped FBS) for 24h. Then cells were treated with siRNASEH2A #1, #2 or siControl for 48h. Protein expression was evaluated by western blot analysis.

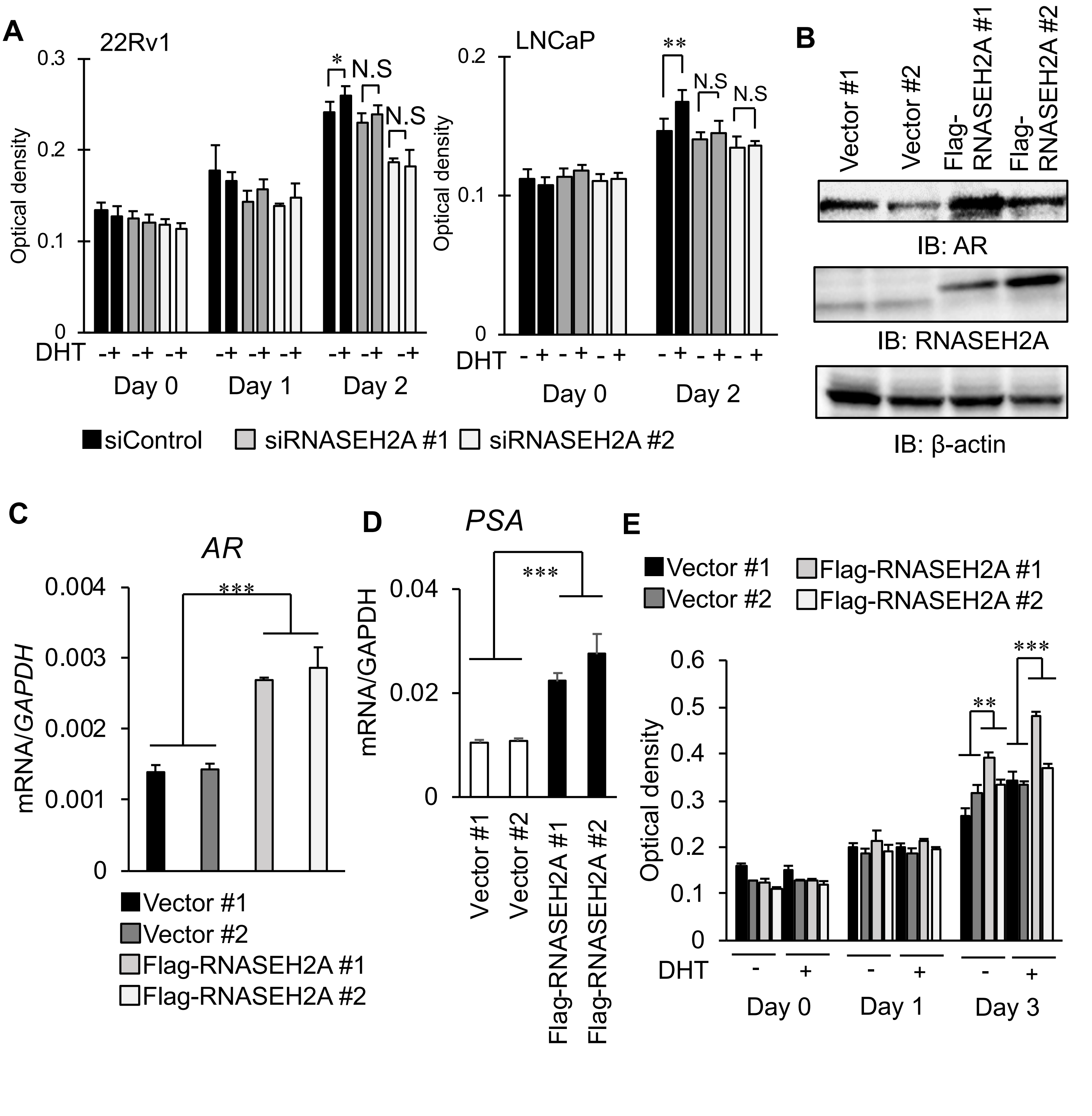

Supplementary Figure S5. RNASEH2A positively regulates AR-mediated cell growth. (A) Evaluation of the effect of siRNASEH2A treatment on the cell proliferation by DHT 10 nM treatment. Cell proliferation was evaluated by MTS assay. Statistical analysis was performed by using of two-sided Student's t-test (N.S: not significant, *: $P < 0.05$). Data represent mean + SD (N = 6). (B) AR protein expression in LNCaP cells stably expressing RNASEH2A. Protein expression was evaluated by western blot analysis. (C) The mRNA expression level of *AR* in LNCaP cells stably expressing RNASEH2A. Statistical analysis was performed by using two-way ANOVA (***: $P < 0.001$). Data represent mean + SD (N = 3). (D) The ratio of upregulation of mRNA expression of *PSA* by using of androgen stimulate in LNCaP cells stably expressing RNASEH2A. DHT 10 nM was added 72h after cell seed. Statistical analysis was performed by using two-way ANOVA (***: $P < 0.001$). Data represent mean + SD (N = 3). (E) Evaluation of the effect of DHT 10 nM treatment on the cell proliferation in LNCaP cells stably expressing RNASEH2A. Cell proliferation was measured by MTS assay. Statistical analysis was performed by using two-way ANOVA (*: $P < 0.05$, **: $P < 0.01$, ***: $P < 0.001$). Data represent mean + SD (N = 6).

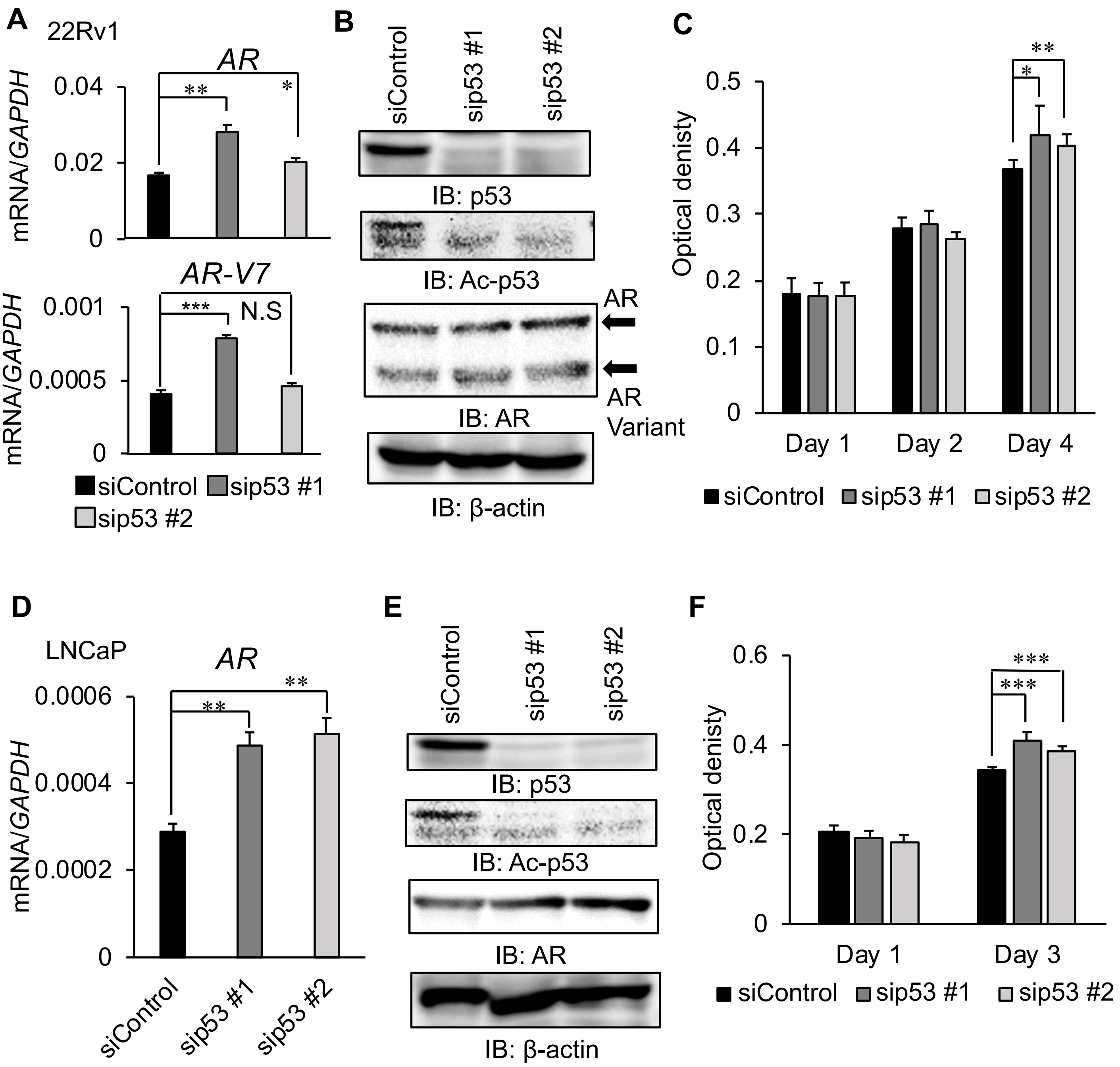

Supplementary Figure S6. Knockdown of p53 induced AR expression and cell growth. (A-C) 22Rv1 was treated with sip53 #1, #2, or siControl. (A) mRNA level of *AR* and *AR-V7* were measured by qRT-PCR. Statistical analysis was performed by using of two-sided Student's t-test (N.S: not significant, *: $P < 0.05$, **: $P < 0.01$, ***: $P < 0.001$). Data represent mean + SD (N = 3). (B) Protein levels of AR, p53, and acetylated-p53 (Ac-p53) were evaluated by western blot analysis. (C) Cell growth was evaluated by MTS assay. Statistical analysis was performed by using of two-sided Student's t-test (*: $P < 0.05$, **: $P < 0.01$). Data represent mean + SD (N = 6). (D-F) LNCaP was treated with sip53 #1, #2, or siControl. (D) mRNA level of *AR* was measured by qRT-PCR. Statistical analysis was performed by using of two-sided Student's t-test (N.S: not significant, *: $P < 0.05$, **: $P < 0.01$, ***: $P < 0.001$). Data represent mean + SD (N = 3). (E) Protein levels of AR, p53, and acetylated-p53 (Ac-p53) were evaluated by western blot analysis. (F) Cell growth was evaluated by MTS assay. Statistical analysis was performed by using of two-sided Student's t-test (***: $P < 0.001$). Data represent mean + SD (N = 6).

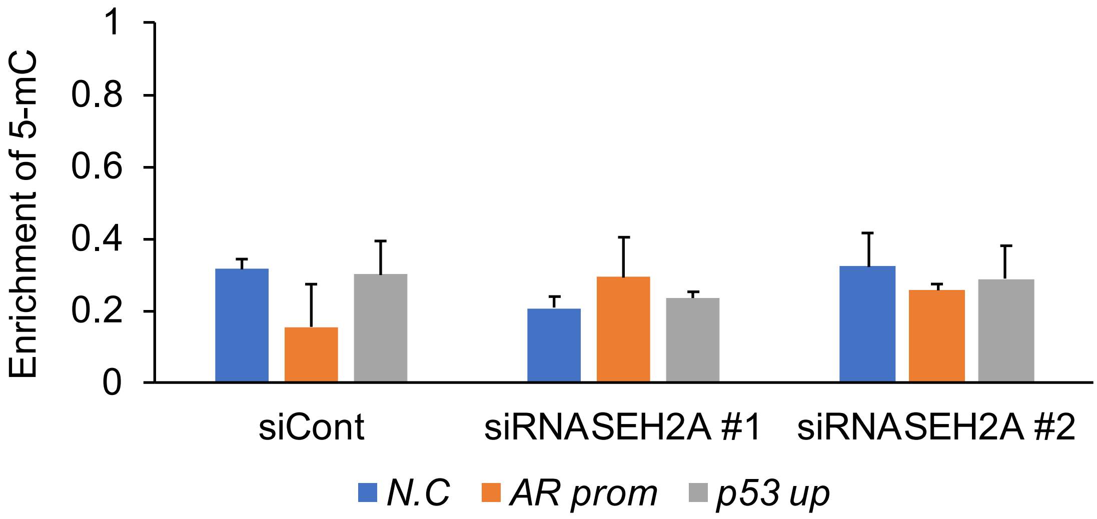

Supplementary Figure S7. DNA methylation is not significantly affected by RNASEH2A knockdown. Fragmented DNA is immunoprecipitated using a monoclonal antibody to 5-mC. The resulting enrichment of methylated DNA in the immunoprecipitated fraction at specific regions (AR prom: AR promoter including CpG island, p53 up: upstream regions of p53 promoter including CpG island.) was determined by qPCR. N.C: negative control

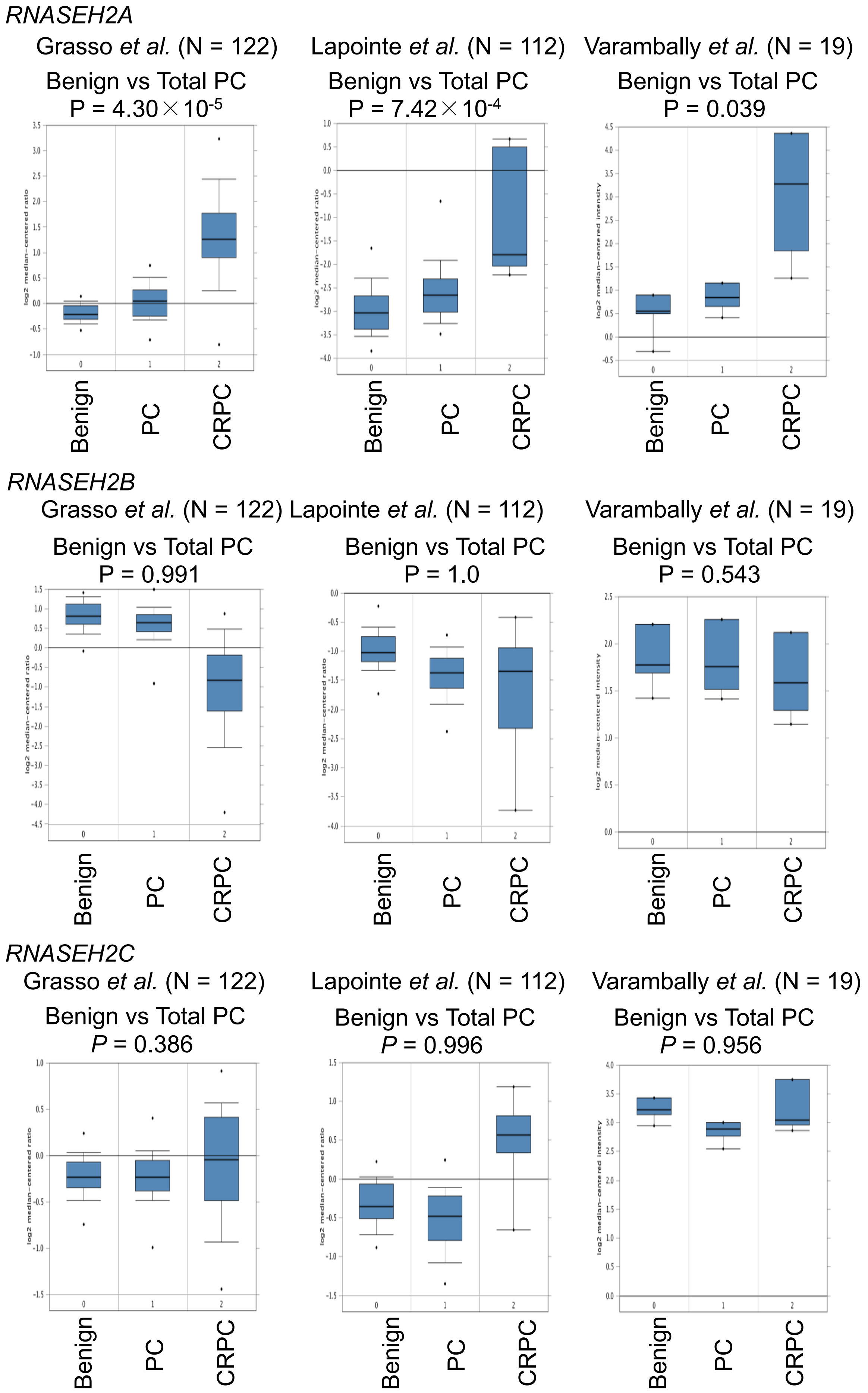

Supplementary Figure S8. Each RNASEH2 subunit gene expression in PC by using public database. mRNA expression of each RNASEH2 subunit by Oncomine. Statistical analysis was performed by using of two-sided Student's t-test. PC: prostate cancer, CRPC: castration-resistant PC.

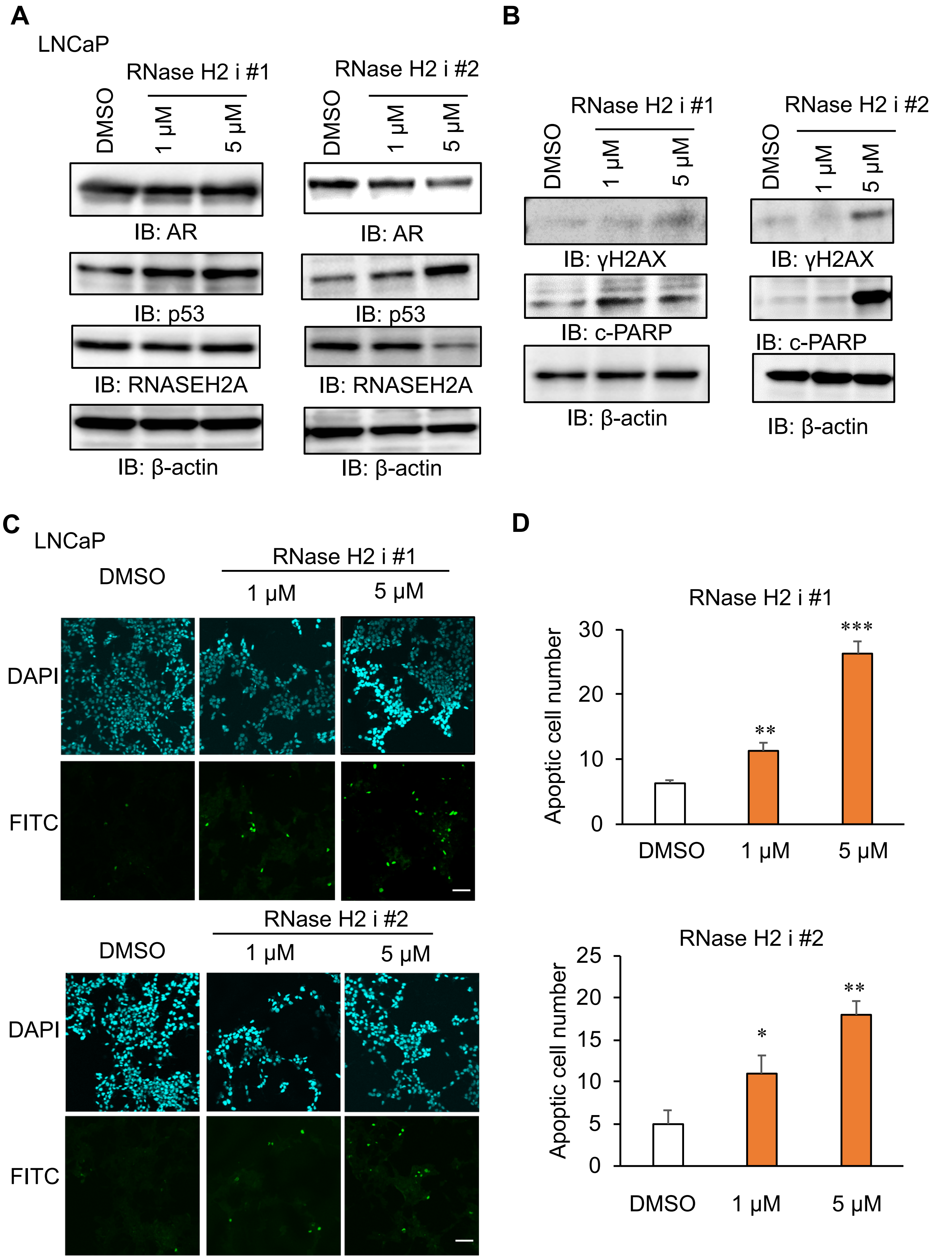

Supplementary Figure S9. The effect of RNase H2 i treatment on LNCaP cells. (A, B) Western blot analysis of the expression of AR, p53, γ-H2AX, and c-PARP. (C, D) TUNEL assay (C) Representative picture of FITC positive cell which was induced by RNase H2 i. (D) TUNEL positive cell was counted at randomly four fields. Figure exhibited by four fields means. Statistical analysis was performed by using of two-sided Student's t-test (*: $P < 0.05$, **: $P < 0.01$, ***: $P < 0.001$). Data represent mean + SD (N = 3).

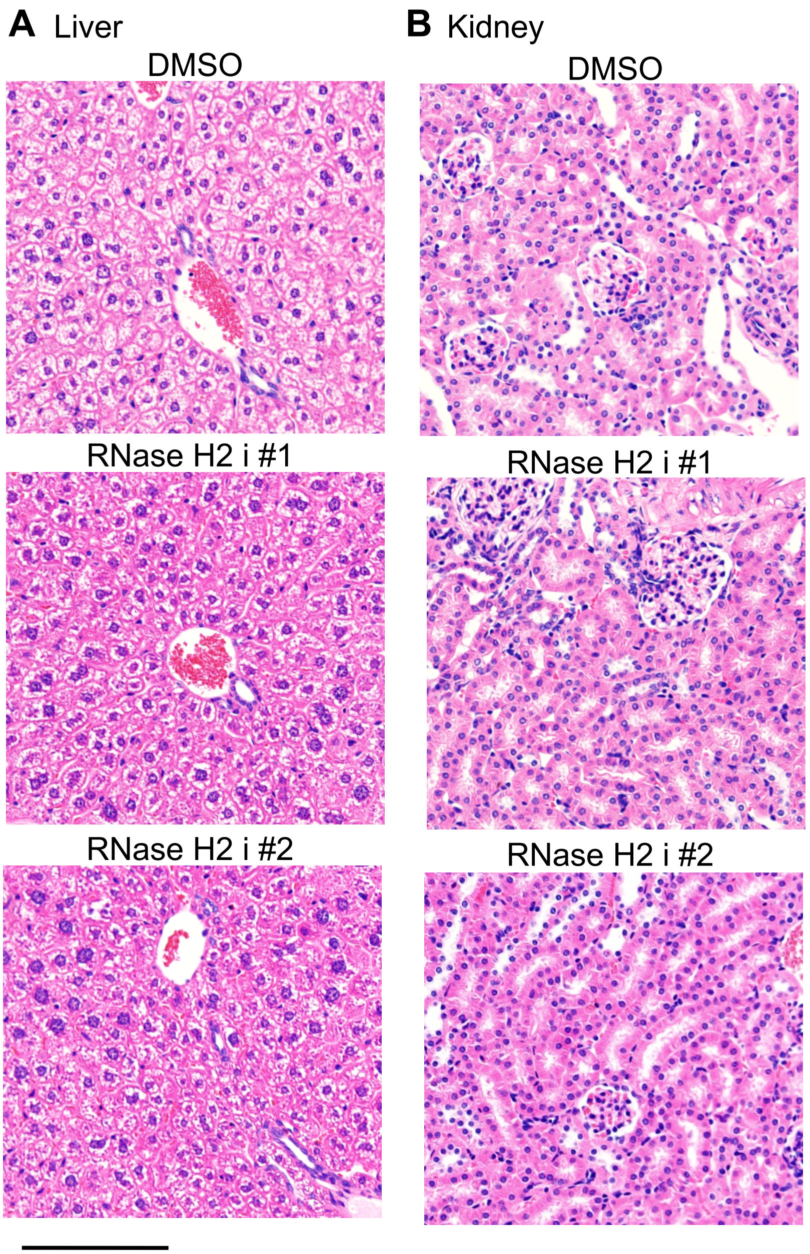

Supplementary Figure S10. No apparent toxic effect was observed in tissues of mice treated with RNase H2 i. Hematoxylin and eosin (HE) staining of liver (A) and kidney (B) in mice harboring 22Rv1 tumors treated with vehicle or RNase H2 i. Bar = 100 µm
